# Identifying potential keystone bacterial species within the phycosphere of marine algae and unveiling their metabolic characteristics

**DOI:** 10.1007/s42995-025-00325-6

**Published:** 2025-10-28

**Authors:** Jeong Min Kim, Byeong Jun Choi, Hülya Bayburt, Jae Kyeong Lee, Che Ok Jeon

**Affiliations:** Department of Life Science, Chung-Ang University, Seoul, 06974 Republic of Korea

**Keywords:** Marine macroalgae, Keystone species, Phycosphere, Microbiome, Network analysis, Metabolic interactions

## Abstract

Metabolic interactions between microbiomes and algal hosts within the phycosphere of marine macroalgae are drawing increasing attention due to their roles in food webs, global nutrient cycles, industries, and their potential as food resources. However, these relations remain poorly understood. In this study, 43 marine macroalgae, including red, brown, and green algae, were collected from the coastal areas of Korea. We identified the bacterial communities within the loosely and tightly attached environments (LAEs and TAEs, respectively) of the phycosphere, along with those in the surrounding seawater, using 16S rRNA gene sequencing. *β*-Diversity analysis revealed significant differences between the bacterial communities among the three, with minimal variation related to sampling location or algal color. Indicator value analysis identified *Pseudoalteromonas* (in the LAE and TAE), *Psychromonas* (in the LAE), *Marinomonas* (in the LAE), and *Litorimonas* (in the TAE) as the dominant taxa in the phycosphere, in contrast to seawater. Network analysis suggested positive correlations among taxa within the same environments and negative correlations between those in the LAE and TAE, highlighting their distinct ecological conditions. Analysis using the Phylogenetic Investigation of Communities by Reconstruction of Unobserved States and Kyoto Encyclopedia of Genes and Genomes pathways revealed functional variations between the phycosphere- and seawater-residing microbes. The microbial taxa–function relationships were assessed through Spearman’s rank-order correlation. Additionally, bacterial species belonging to the core taxa were isolated and their genomes sequenced. Their metabolic traits were analyzed via bioinformatics, recognizing key metabolic features essential for symbiotic interactions with algal hosts and survival within the phycosphere. The findings of this study advance our understanding of the marine algal phycosphere microbiome by detailing the metabolic characteristics of potential keystone species.

**Supplementary Information:**

The online version contains supplementary material available at 10.1007/s42995-025-00325-6.

## Introduction

Marine algae, including red (Rhodophyta), brown (Phaeophyta), and green (Chlorophyta) algae, comprise ~ 12,000 diverse species and serve as essential primary producers in the oceans (Lu et al. 2023). They have attracted increasing amounts of attention due to their ecological role in stabilizing marine environments, contributing to global nutrient cycles, as well as for their value as food, food additives, and industrial resources (de Barros Medeiros et al. 2022). In particular, they are known for their rapid growth rates and significant contributions to biological productivity, playing a major role in coastal ecosystems and aquaculture (Sudhakar et al. 2018). As a result, considerable efforts have been dedicated to preserving marine macroalgal ecosystems and cultivating them for commercial purposes (Kim et al. 2017).

The surfaces of macroalgae, known as the “phycosphere,” are colonized by bacteria that have co-evolved with their algal hosts due to complex and robust relationships over time (Bengtson et al. 2017). Recent studies suggest that these associations generally promote algal growth and development, although they can be neutral or even harmful depending on the bacterial species involved (Amin et al. 2015; Cirri and Pohnert 2019). Marine macroalgae provide habitat and nutrients to these bacteria, which in turn protect the algae from pathogens, supply essential nutrients that the hosts cannot synthesize, and stimulate their growth (Alsufyani et al. 2020; Li et al. 2022; Wichard 2023). Given the ecological, economic, and biotechnological importance of macroalgae, numerous studies have been conducted in recent years to better understand the macroalgae–bacteria interactions, although many of them have focused on specific macroalgal species (Saha et al. 2024). In particular, the green macroalgal genus *Ulva* has been studied extensively, thus establishing a model system for exploring algal–bacterial interactions (Califano et al. 2020; Spoerner et al. 2012; Wichard 2023). Distinct sets of core bacterial taxa colonize *Ulva* and form mutualistic relationships that support development, enhance nutrient acquisition, and improve stress tolerance in algae (Califano et al. 2020; KleinJan et al. 2023; Spoerner et al. 2012; Wichard 2023). For instance, specific bacterial strains release morphogenetic compounds essential for normal thallus formation in *Ulva mutabilis* (Spoerner et al. 2012; Ghaderiardakani et al. 2024).

However, these findings primarily reflect interactions observed in a few algal species, and macroalgae–bacteria relations may vary substantially across algal taxa. Consequently, the current knowledge remains insufficient for comprehensively understanding such interactions in varied marine ecosystems. To address this gap, it is crucial to recognize the common metabolic traits of microbial communities and their interactions within the phycosphere of diverse macroalgal species, highlighting the need to identify the core species. In recent years, several studies have focused on identifying key bacterial taxa that are frequently associated with a wide range of marine algae and play critical roles in algal–microbial interactions (Park et al. 2022; Lu et al. 2023; King et al. 2023; Kim et al. 2024).

Although the bacterial communities within the phycosphere vary with the macroalgal phylogeny (algal color) (Bondoso et al. 2017; Lachnit et al. 2009; Nahor et al. 2024), certain bacterial taxa appear to be species-specific (Ihua et al. 2020). Thus, we hypothesize that a subset of bacterial taxa forms a core group of microbiota consistently associated with diverse macroalgal species. These taxa are expected to possess certain conserved metabolic features that contribute to broad interactions with a wide range of marine macroalgae. To test this hypothesis, we collected several marine macroalgal species, including red, brown, and green algae, from three geographically distinct coastal regions. We analyzed the community structures of these bacteria associated with the algal phycosphere and conducted a comprehensive statistical analysis to identify common core bacterial taxa with the potential to act as keystone species. Furthermore, we isolated the bacterial species corresponding to these taxa and investigated their metabolic traits, which were related to algal interactions, using genome-based bioinformatic analyses.

## Materials and methods

### Collection of marine algae and sample preparation for community analysis

Marine algae and seawater were collected in sterilized zipper bags from six sites (two per coast) along the South, West, and East Seas of the Korean Peninsula between April 26 and 27, 2023 (Fig. S1). Algal taxonomy was determined by sequencing a fragment of the ribulose-1,5-bisphosphate carboxylase/oxygenase (*rbcL*) gene following Wawrik et al. (2002). Algal thalli were lightly rinsed with seawater, filtered through a 0.2 µm filter, and cut into 2–3 cm pieces with sterilized scissors.

Bacterial communities associated with the loosely and tightly attached environments (LAEs and TAEs, respectively) of algae were analyzed separately. For LAEs, five tissue pieces from different regions of each alga were pooled and vigorously vortexed for 10 s in a 10 mL conical tube containing 3 mL of artificial seawater (ASW; Lee et al. 2024). The liquid fraction was centrifuged at 16,100 × *g* for 5 min at 4 °C, and the resulting pellets were used for analysis. For TAEs, the remaining algal tissues were gently vortexed with 3 mL of ASW for 10 s to remove residual LAE bacteria, and the liquid was discarded. The tissues were then homogenized in 3 mL of ASW using a T10 basic homogenizer (IKA Werke GmbH & Co. KG, Germany), centrifuged at 16,100 ×*g* for 5 min, and the pellets were analyzed. For seawater communities, 4 L of seawater collected near algae at each site was sequentially filtered through 5.0 and 0.22 µm nylon membranes (Millipore, USA). Both filters were cut into small pieces with sterile scissors and processed for community analysis.

### Bacterial community analysis

Genomic DNA was extracted from LAE and TAE pellets and from membrane filter fragments using the FastDNA^™^ SPIN Kit (MP Biomedicals, CA, USA) according to the manufacturer’s instructions. The hypervariable V3–V4 regions of the bacterial 16S rRNA gene were PCR-amplified, sequenced on an Illumina MiSeq platform (USA), and analyzed with the QIIME2 v2023.5 pipeline (Bolyen et al. 2019) as previously described (Baek et al. 2023). Briefly, sequencing reads were demultiplexed by barcode, with barcodes and adapters trimmed using Cutadapt (https://cutadapt.readthedocs.io/en/stable/). Quality filtering (threshold ≤ 25), denoising, and paired-end merging were performed with the DADA2 v2023.5 plugin (http://benjjneb.github.io/dada2/), and chimeric and singleton amplicon sequence variants (ASVs) were removed. High-quality reads were used to calculate *α*-diversity metrics (operational taxonomic units, Shannon–Weaver, Chao1 richness, and Simpson indices) within QIIME2. Taxonomic classification of ASVs was conducted using the QIIME2 “feature-classifier” plugin and the classify-sklearn method with the SILVA 138 reference database (silva-138-99-nb-classifier). Results at the genus level were visualized as bubble plots, excluding reads assigned to mitochondrial or chloroplast 16S rRNA sequences. Relative abundances across red, brown, and green algae were displayed as box plots in R using “ggplot2”, and intergroup differences were tested with the Wilcoxon rank-sum test.

### Statistical analyses of the bacterial communities

An algal phylogenetic tree based on *rbcL* sequences was constructed using the Neighbor-Joining algorithm in MEGA11 (Tamura et al. 2021). Bacterial communities in the LAE and TAE were hierarchically clustered using the Bray–Curtis dissimilarity metric, and the qiime diversity beta-rarefaction command in QIIME2 (https://amplicon-docs.qiime2.org). Comparisons among seawater, LAE, and TAE communities were performed using Bray–Curtis dissimilarities in the “phyloseq” package in R (https://joey711.github.io/phyloseq/) and visualized with a non-metric multidimensional scaling (NMDS) plot. *β*-Diversity dissimilarities across algal groups (red, brown, and green), collection sites (South, West, and East Seas), and attachment types (seawater, LAE, and TAE) were statistically tested with PERMANOVA (999 permutations) using the pairwise adonis function in the “vegan” package (v2.6-4) in R (https://cran.r-project.org/web/packages/vegan/index.html). To identify indicator taxa distinguishing seawater, LAE, and TAE, Indicator Value (IndVal) analysis was performed on genus-level relative abundances with the *multipatt* function in the “indicspecies” package in R (Cáceres and Legendre 2009).

Ecological correlation networks among bacterial taxa in seawater, LAE, and TAE were constructed from genus-level relative abundances using the Sparse Correlations for Compositional Data (SparCC) method in the NetCoMi package (v1.0.3; Peschel et al. 2021). Co-occurrence networks showing significant correlations (|*r*|> 0.2; *p* < 0.05) were visualized in Cytoscape (https://cytoscape.org/).

### Phylogenetic investigation of communities by reconstruction of unobserved states (PICRUSt) and Spearman’s rank-order correlation analyses

Metabolic functions of bacteria in seawater, LAE, and TAE were predicted from 16S rRNA gene sequences using PICRUSt2 v2.5.2 (Douglas et al. 2020). Predicted functional genes were assigned to KEGG pathways based on KEGG Orthology (KO) using the “ggpirust2” package in R (Yang et al. 2023). Relative abundances of KEGG pathways were assessed at secondary and tertiary levels, and significant differences among environments were tested with Welch’s t-test. Correlations between bacterial taxa and KEGG functional genes at the tertiary level were evaluated with Spearman correlation analysis in R, and significant positive correlations (*r* > 0.3; *p* < 0.05) were visualized as networks in Cytoscape.

### Isolation of core species from the phycospheres, genome sequencing, and bioinformatic analysis

Bacterial strains identified as core taxa within phycospheres by IndVal analysis were isolated from algal homogenates used for TAE community analysis. The homogenates were serially diluted in ASW, spread onto marine agar (MA; MBcell, South Korea), and incubated aerobically at 25 °C. The colonies were selected, and their 16S rRNA genes were amplified and digested with HaeIII and HhaI as described by Lee et al. (2024). Products with fragment patterns matching the target taxa were sequenced, and phylogenetic classification was conducted using the Nucleotide Similarity Search program (http://www.ezbiocloud.net/identify/; Yoon et al. 2017).

For genome sequencing, isolates identified as core taxa were cultured in marine broth (MBcell). Genomic DNA was extracted with the Wizard Genomic DNA Purification Kit (Promega, USA) and sequenced on both the Illumina HiSeq X (151 bp paired-end; Illumina, USA) and MinION (Oxford Nanopore Technologies) platforms. Long reads were de novo assembled with Flye v2.9.3 (Kolmogorov et al. 2019) and iteratively polished with Illumina reads using Pilon v1.24 (Walker et al. 2014) until no further exclusions were required. For phylogenomic analysis, sequences of 120 bacterial marker proteins were extracted from the genomes of the isolates and related type strains, aligned using GTDB-Tk (Chaumeil et al. 2020), and used to construct Maximum-Likelihood trees in MEGA11. Average nucleotide identity (ANI) and digital DNA-DNA hybridization (dDDH) values were calculated with the OrthoANI tool (Lee et al. 2016) and the Genome-to-Genome Distance Calculator v3.0 (Meier-Kolthoff et al. 2013), respectively.

Genome sequences were deposited in GenBank. Protein sequences predicted by the NCBI Prokaryotic Genome Annotation Pipeline were functionally annotated against the KO database using BlastKOALA (Kanehisa et al. 2016). Genes associated with symbiosis and survival in phycospheres were further validated by BLASTP searches against UniProt reference proteins (https://www.uniprot.org). Secondary metabolite biosynthetic gene clusters were identified with antiSMASH 7.0 (Blin et al. 2023), and carbohydrate-active enzyme (CAZyme) genes were detected using DIAMOND and HMMER searches against the dbCAN3 database (Zheng et al. 2023).

## Results

### Collection and taxonomic identification of marine macroalgae

Marine macroalgae were collected from the South, West, and East Seas of the Korean Peninsula and identified taxonomically based on their *rbcL* sequences. Macroalgae from the same collection site that were phylogenetically identical were excluded from bacterial community analysis. In total, 26 red, 7 brown, and 10 green algae were selected (Table S1). These samples represented a wide range of marine macroalgae from Rhodophyta, Phaeophyta, and Chlorophyta for analyzing the bacterial communit*y* structure in the LAE and TAE regions.

### Analysis of the bacterial community in the LAE and TAE of marine macroalgae and seawater

To identify the bacteria that may exhibit symbiotic relationships with marine macroalgae, we statistically analyzed the *α*-diversity indices and bacterial communities in the LAE and TAE pellets of 43 macroalgae and six seawater samples (Tables S2, S3, and S4). The Chao1 index, which estimates species richness, showed that the bacterial diversity was lower in TAE (248.1 ± 130.5) and LAE (170.4 ± 86.1) compared to seawater (355.9 ± 256.5). This pattern is most likely attributable to the more stable environmental conditions in the phycosphere compared to the dynamic seawater environment. Furthermore, Chao1 indices for LAE (131.4 ± 98.8) and TAE (184.4 ± 61.2) from the West Sea were lower than those from the South Sea (LAE: 163.3 ± 61.6; TAE: 311.1 ± 152.8) and East Sea (LAE: 225.8 ± 81.3; TAE: 203.0 ± 79.8). Shannon–Weaver indices, which reflect species evenness, remained relatively consistent across attachment types (LAE, TAE, and seawater), algal type (red, brown, and green), and collection locations (South, West, and East Seas).

**Fig. 1:**
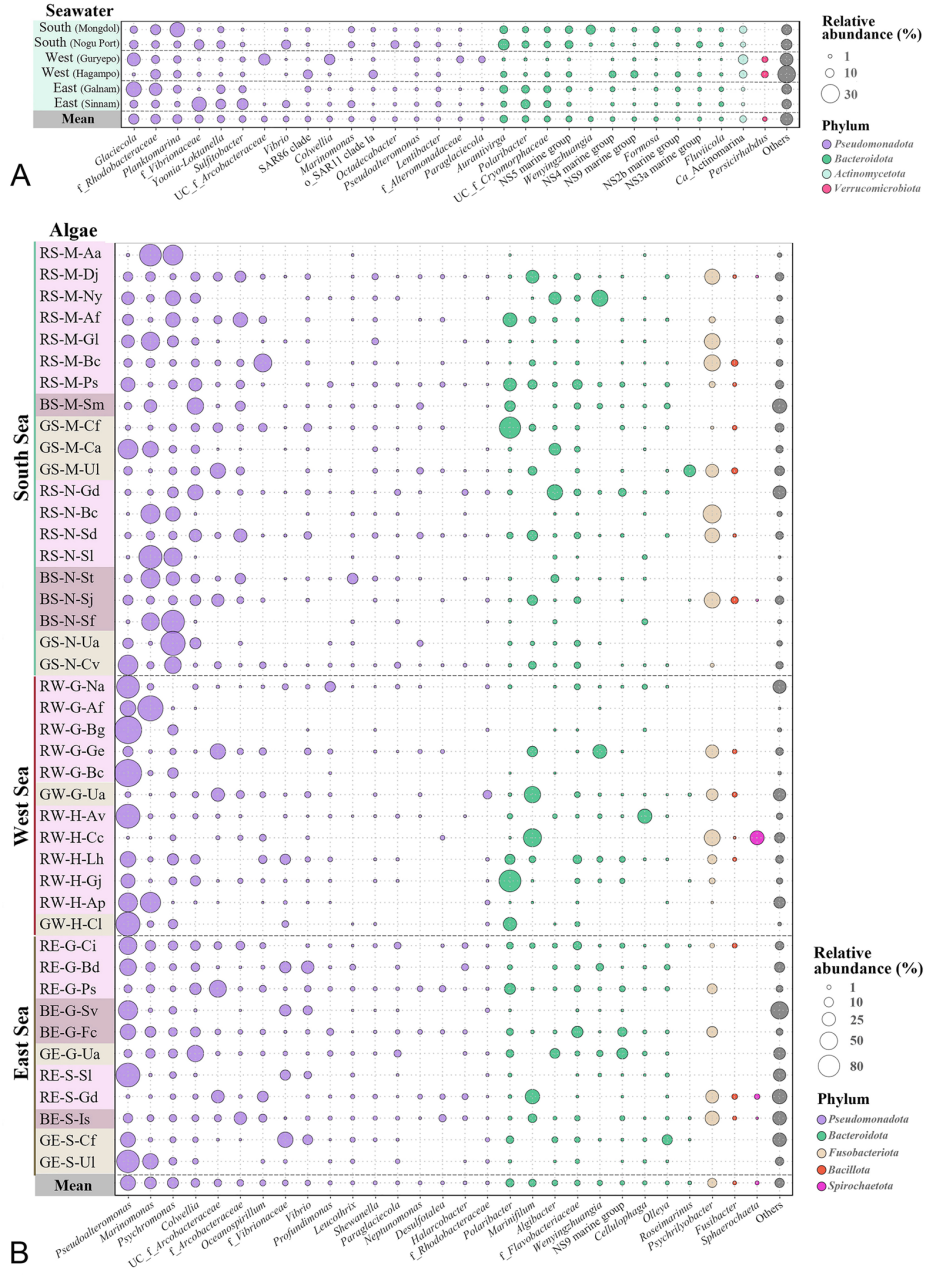
Genus-level bacterial communities associated with the marine algae collected from the seawater (**A**) and from the loosely attached environment (LAE; **B**). The “Others” category includes bacterial taxa with relative abundances ≤ 0.6% in the seawater and ≤ 0.3% in all LAE samples. In panel B, the left column lists the names of the algal strains (Table S1), with bacterial genera belonging to different phyla shown in distinct colors

**Fig. 2:**
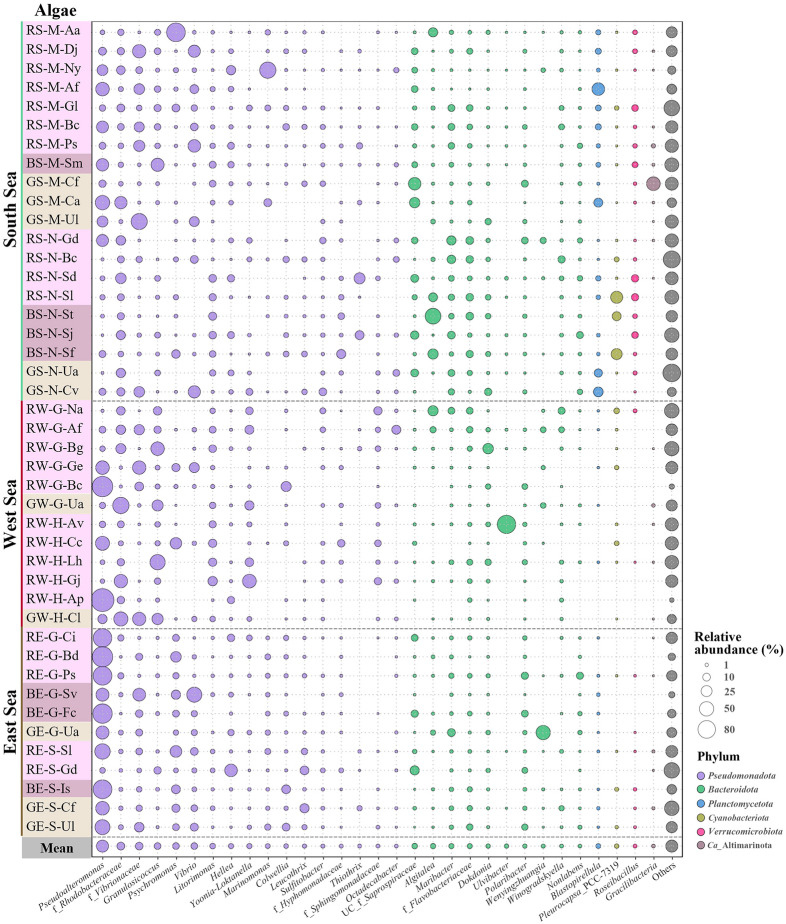
Genus-level bacterial communities from the tightly attached environment (TAE) of marine algae. The “Others” category includes bacterial taxa with relative abundances ≤ 0.6% in all TAE samples. The left column lists the names of the algal strains (Table S1), with bacterial genera belonging to different phyla shown in distinct colors

Community analysis revealed distinct bacterial compositions between the seawater, LAE, and TAE samples. In seawater, *Glaciecola,* f_*Rhodobacteraceae*, *Planktomarina*, f*_Vibrionaceae*, *Yoonia-Loktanella*, *Sulfitobacter*, UC_f*_Arcobacteraceae*, *Aurantivirga*, and *Polaribacter* were abundant, and their profiles varied significantly by collection site despite all sites being open to the same ocean (Fig. 1A). Common genera across all seawater samples included *Glaciecola*, *Planktomarina*, *Yoonia-Loktanella*, *Sulfitobacter*, *Aurantivirga*, and *Polaribacter*, whereas *Vibrio*, *Colwellia*, *Marinomonas*, *Wenyingzhuangia*, and *Persicirhabdus* were identified only in certain samples*.* In contrast, the LAE samples exhibited marked variations depending on the algae, with *Pseudoalteromonas*, *Marinomonas*, *Psychromonas*, *Colwellia*, UC_f_*Arcobacteraceae*, f_*Arcobacteraceae*, *Oceanospirillum*, *Vibrio*, *Polaribacter*, *Marinifilum*, and *Algibacter* being the most abundant (Fig. 1B). Notably, *Pseudoalteromonas*, *Psychromonas*, *Marinifilum*, and *Psychrilyobacter*, which were minor or absent in seawater, were prevalent in LAE. Additionally, Fusobacteriota and Spirochaetota, which were absent in seawater, were abundant in a few LAE samples. In the TAE samples, *Pseudoalteromonas*, f*_Rhodobacteraceae*, f*_Vibrionaceae*, *Granulosicoccus*, *Psychromonas*, *Vibrio*, *Litorimonas*, UC_f_*Saprospiraceae*, *Algitalea*, *Maribacter*, *Dokdonia*, *Polaribacter*, and *Blastopirellula* were markedly prominent, with significant variations depending on the algae (Fig. 2). Notably, *Hellea*, *Granulosicoccus*, *Algitalea*, *Cyanobacteria*, and *Gracilibacteria*, which were absent in seawater and LAE, were found only in certain TAE samples. Conversely, *Fusobacteriota* and *Spirochaetota*, present in some LAE samples, were not detected in TAE. These findings indicate that LAE and TAE regions, which may harbor symbiotic bacteria, possess distinct bacterial communities compared to seawater. Moreover, the community composition varied remarkably among specific macroalgae, though no distinct genera were found based on algal type or collection site.

### Comparison of bacterial communities according to algal phylogeny, collection site, and attachment

**Fig. 3:**
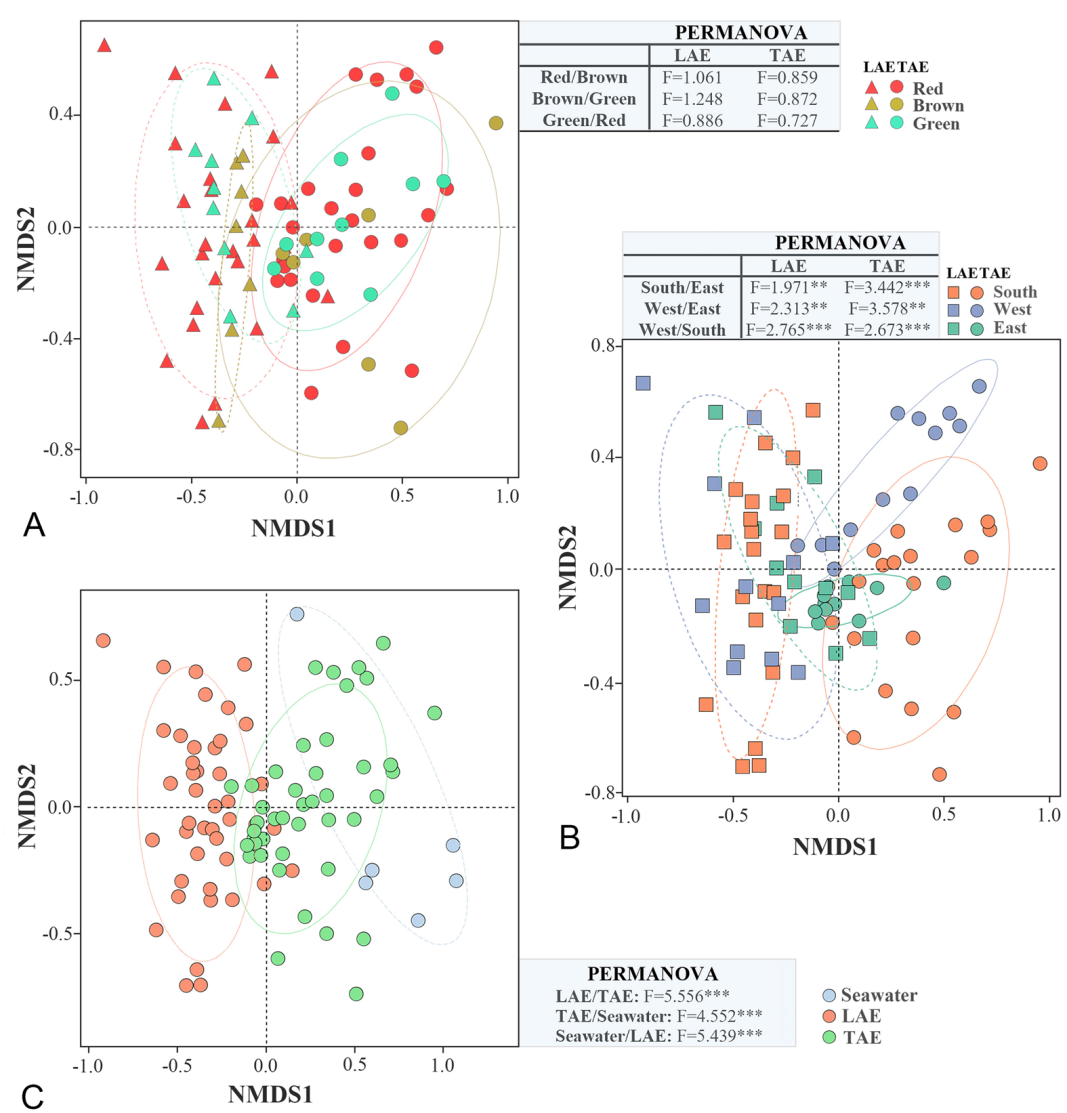
NMDS plots comparing bacterial communities across samples: red, brown, and green algae (**A**); South, West, and East Seas (**B**); and LAE, TAE, and seawater (**C**). Community dissimilarities were calculated using the Bray–Curtis metric. The statistical significance was assessed by PERMANOVA, with panels A and B analyzed separately for LAE and TAE. **p < 0.01 and ***p < 0.001

Given that microbial communities are believed to be shaped by the metabolic traits of the host marine algae, we examined the relationship between marine algal phylogeny and the bacterial communities within the LAE and TAE; however, no correlation was observed (Fig. S2). Box plot analysis of the core taxa shared across algal hosts revealed no significant variations in their relative abundances among algal types (red, brown, and green) in the LAE and TAE (Fig. S3). Such a lack of any correlation was further confirmed by NMDS analysis, which revealed that bacterial communities within the LAE and TAE could not be distinctly differentiated based on algal phylogeny (algal type), as supported by the findings of the PERMANOVA analysis (Fig. 3A). The bacterial communities also did not reveal any significant differentiation based on algal phylogeny in all macroalgal groups collected from the South, West, and East Seas (Fig. S4). In contrast, NMDS analysis demonstrated marked variations in bacterial communities based on collection site (South, West, and East Seas) in LAE and TAE (Fig. 3B). Additionally, the bacterial communities associated with red macroalgae exhibited a certain degree of variability by collection site (South vs. East in TAE; West vs. East and West vs. South in LAE and TAE; Fig. S5). However, bacterial communities associated with the brown and green macroalgae did not show any remarkable differentiation by collection site, most likely due to the small sample sizes. These findings indicated that the bacterial communities within the phycosphere of macroalgae were more distinctly influenced by the collection site than by algal phylogeny. Additionally, bacterial communities were differentiated by attachment type (Fig. 3C), with a particularly strong separation between LAE and TAE (*F* = 5.556; *p* < 0.001). Given that bacteria associated with marine algae are more tightly attached, we focused on identifying the bacterial species driving the variation among the seawater, LAE, and TAE communities to identify those that are most likely to engage in interactions with marine algae.

### Identification of putative core bacterial taxa potentially associated with macroalgal phycospheres

**Fig. 4:**
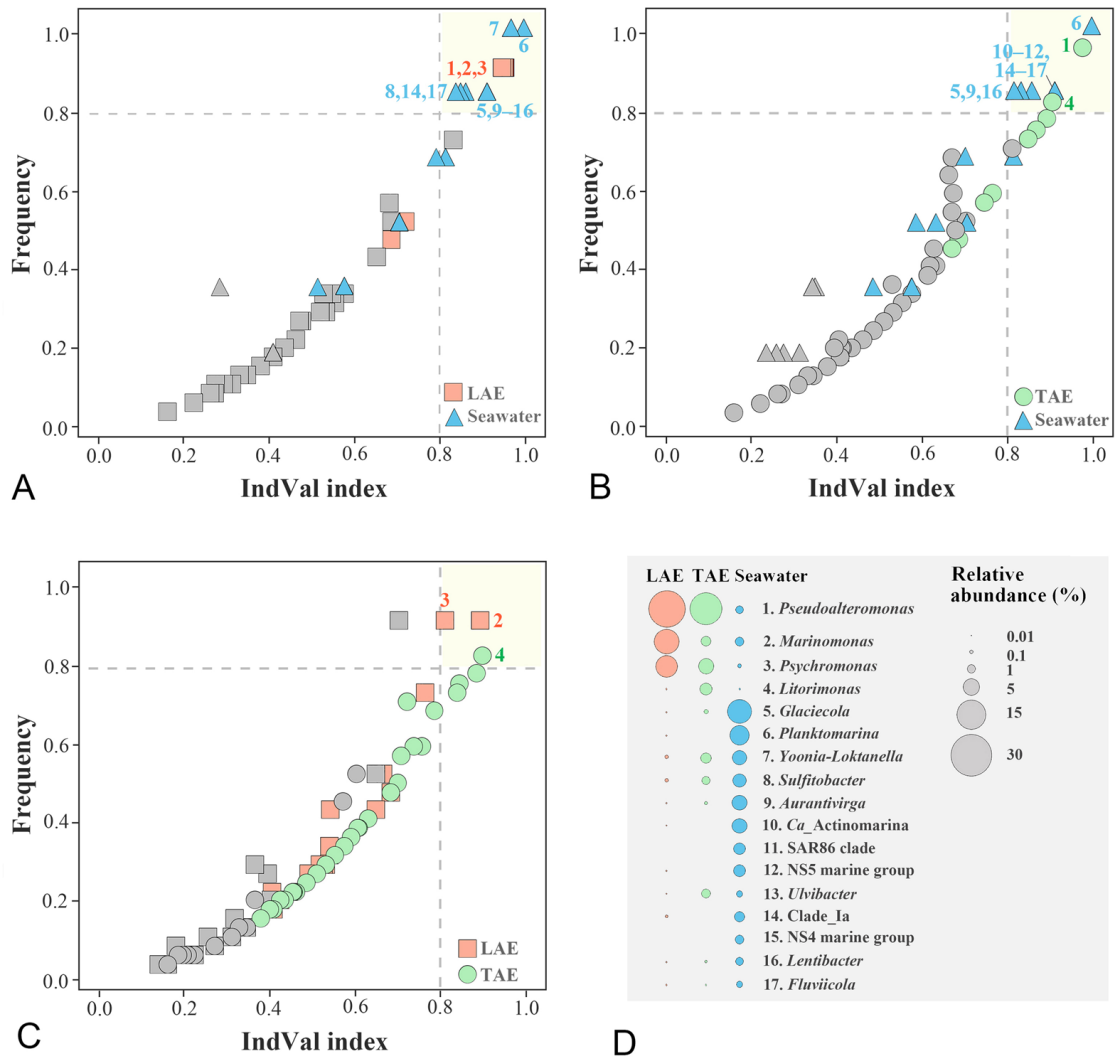
IndVal analysis identifying the bacterial genera that differ among the LAE, TAE, and seawater communities: seawater versus LAE (**A**), seawater versus TAE (**B**), and LAE versus TAE (**C**). Genera with IndVal and frequency > 0.8 are highlighted in light yellow, with their mean relative abundances within each environment shown in **D**. In A–C, taxa with no statistically significant differences (*p* > 0.05) are indicated in gray

To identify the putative core bacterial taxa within the phycosphere with potential involvement in interactions with macroalgae, we performed an IndVal analysis to identify the bacterial species that differentiated the LAE- and TAE-associated communities from those in seawater, along with assessing their frequencies in these communities. Between LAE and seawater, *Pseudoalteromonas, Psychromonas*, and *Marinomonas* exhibited an IndVal index and frequency > 0.8 in LAE (Fig. 4A). Similarly, between TAE and seawater, *Pseudoalteromonas* and *Litorimonas* showed an IndVal index and frequency > 0.8 in TAE (Fig. 4B), suggesting that *Pseudoalteromonas* may be a core taxon specific to LAE and TAE but not seawater. *Marinomonas* and *Psychromonas* or *Litorimonas* were identified as indicator species for LAE or TAE, respectively (Fig. 4C). These findings are further supported by the evidently higher abundances of *Pseudoalteromonas, Psychromonas, Marinomonas,* and *Litorimonas* in LAE and/or TAE compared to seawater (Fig. 4D), indicating that these genera are most likely the core taxa involved in interactions with marine algae.

**Fig. 5:**
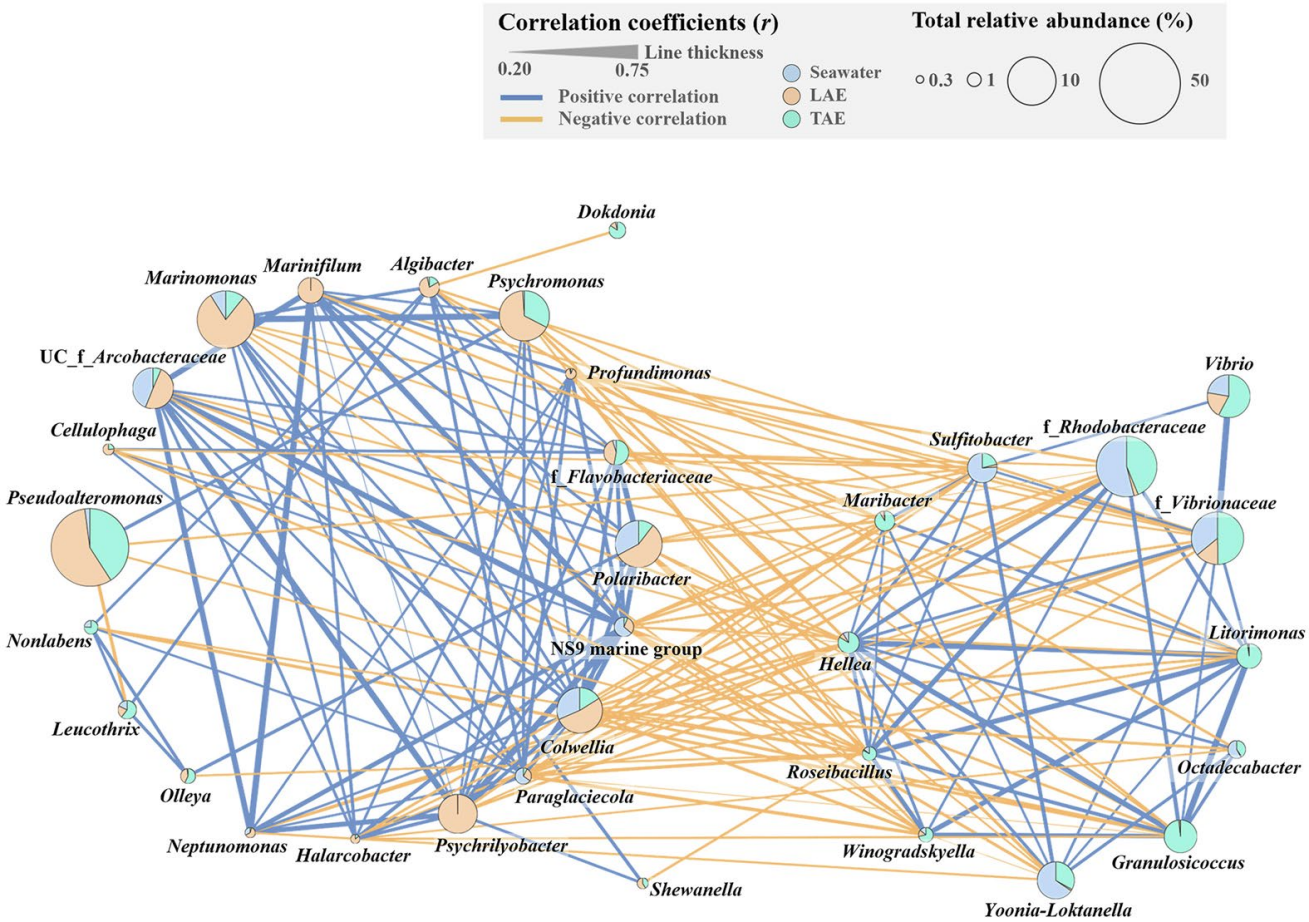
Ecological correlation networks showing the co-occurrence patterns of bacterial taxa in the seawater, LAE, and TAE, as analyzed using the SparCC method. Pie charts display the total mean relative abundances of bacterial taxa, with segments representing their distribution across environments. Edges indicate statistically significant correlations: positive (*r* > 0.2, *p* < 0.05; blue) and negative (*r* <  − 0.2, *p* < 0.05; orange). The line thickness was proportional to the correlation strength

To explore the ecological relationships among bacterial taxa in the seawater, LAE, and TAE, co-occurrence networks were analyzed with SparCC. It identified 33 nodes (bacterial genera) and 186 edges (representing positive or negative correlations with *r* <  ± 0.2, *p* < 0.05) within the bacterial communities (Fig. 5). As expected, the bacterial taxa that were distinctly abundant in each environment (LAE, TAE, and seawater) generally exhibited positive correlations with other taxa within the same environment. For example, LAE-associated taxa, including *Marinomonas*, *Psychromonas*, *Psychrilyobacter*, *Marinifilum*, *Algibacter*, and *Profundimonas*, were positively correlated with the other LAE-associated taxa. Similarly, TAE-associated taxa such as *Litorimonas*, *Granulosicoccus*, *Hellea*, *Maribacter*, and *Roseibacillus* were positively correlated with the other taxa within TAE. However, the LAE- and TAE-associated taxa typically displayed negative correlations with each other, signifying the distinct environmental conditions of LAE and TAE and suggesting metabolic interactions within these environments. Meanwhile, *Pseudoalteromonas*, identified as a core taxon in LAE and TAE, demonstrated a few positive or negative correlations with other bacterial taxa, suggesting that its ability to thrive independently may help its establishment as a core taxon in both.

### Comparison of the predicted metabolic functions of bacterial communities in the seawater, LAE, and TAE

**Fig. 6:**
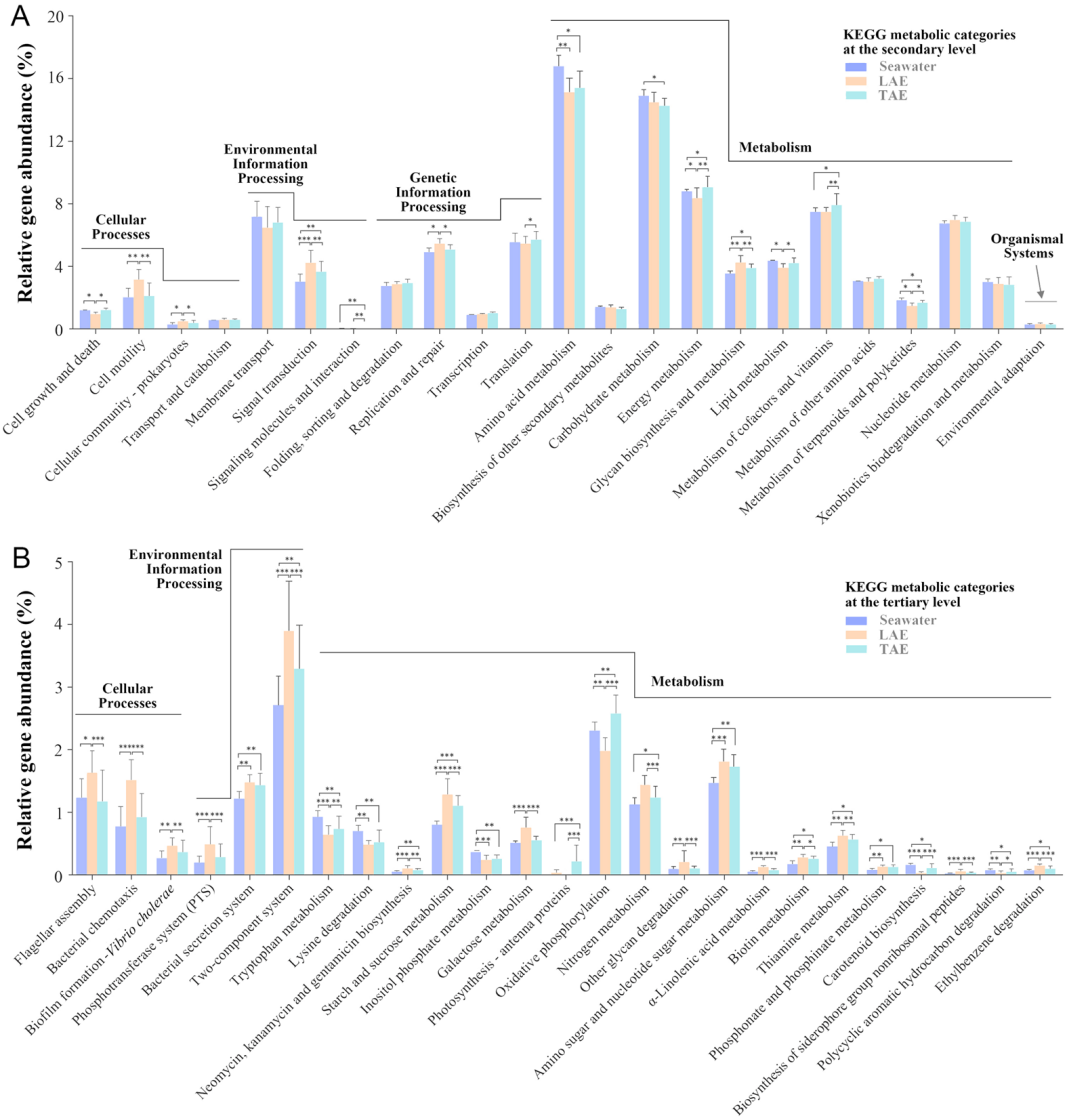
Abundance profiles of the KEGG functional genes in bacteria from the seawater, LAE, and TAE predicted using PICRUSt2, shown at the secondary (**A**) and tertiary (**B**) levels. Panel A displays the representative KEGG categories at the second level, while panel B shows only those at the third level with statistically significant differences. *p < 0.05, **p < 0.01, and ***p < 0.001

The metabolic and functional features of bacterial communities in the seawater, LAE, and TAE were predicted using PICRUSt2. The differences were compared based on KEGG functional category gene abundances (Fig. 6). At the secondary KEGG level, functional distributions were generally similar across the three environments. However, genes associated with “cell motility,” “cellular community-prokaryotes,” “signal transduction,” “signaling molecules and interactions,” and “metabolism of cofactors and vitamins,” which are likely related to symbiotic relationships, were markedly more abundant in LAE and/or TAE than in seawater (Fig. 6A). This trend suggests that bacterial taxa better suited for symbiotic interactions are more competitive under high-density environments or the attached growth conditions of LAE and TAE.

At the tertiary KEGG level, functional differences between seawater, LAE, and TAE became more prominent, particularly in LAE and TAE (Fig. 6B). Genes related to “flagellar assembly,” “bacterial chemotaxis,” “two-component system,” “biofilm formation-*Vibrio cholerae*,” and “bacterial secretion system,” most likely involved in facilitating adaptation to environmental changes or interactions with marine algae, were more abundant in LAE and/or TAE compared to seawater. This finding indicates that the bacterial taxa in these environments are better adapted to interact with marine algae and respond to environmental changes. Additionally, the genes related to “phosphotransferase system (PTS),” “starch and sucrose metabolism,” “galactose metabolism,” “other glycan degradation,” and “amino sugar and nucleotide sugar metabolism,” were more prevalent in LAE and TAE, particularly in LAE, suggesting an enhanced capacity to utilize carbohydrates from marine algae. Furthermore, genes related to “biotin metabolism,” “thiamine metabolism,” “phosphonate and phosphinate metabolism,” and “biosynthesis of siderophore group nonribosomal peptides,” which may enhance marine algae growth (Kim et al. 2024; Yao et al. 2019), were more abundant in LAE and TAE than in seawater. These findings suggest that bacterial taxa in the phycosphere of marine algae engage in symbiotic interactions that may support algal growth and metabolism.

In contrast, genes related to “lysine degradation,” “tryptophan metabolism,” and “inositol phosphate metabolism” were less abundant in LAE and TAE, most likely due to their rapid consumption by high-density-growth microbes and algae within the phycospheres. Genes involved in “biosynthesis of neomycin, kanamycin, and gentamicin” were more prevalent in LAE and TAE, indicating that the bacterial taxa in these environments may have developed an enhanced antibiotic production ability to compete within dense microbial communities. Additionally, genes associated with “photosynthesis-antenna proteins” were more prevalent in TAE, while genes related to “carotenoid biosynthesis” were more abundant in seawater.

**Fig. 7:**
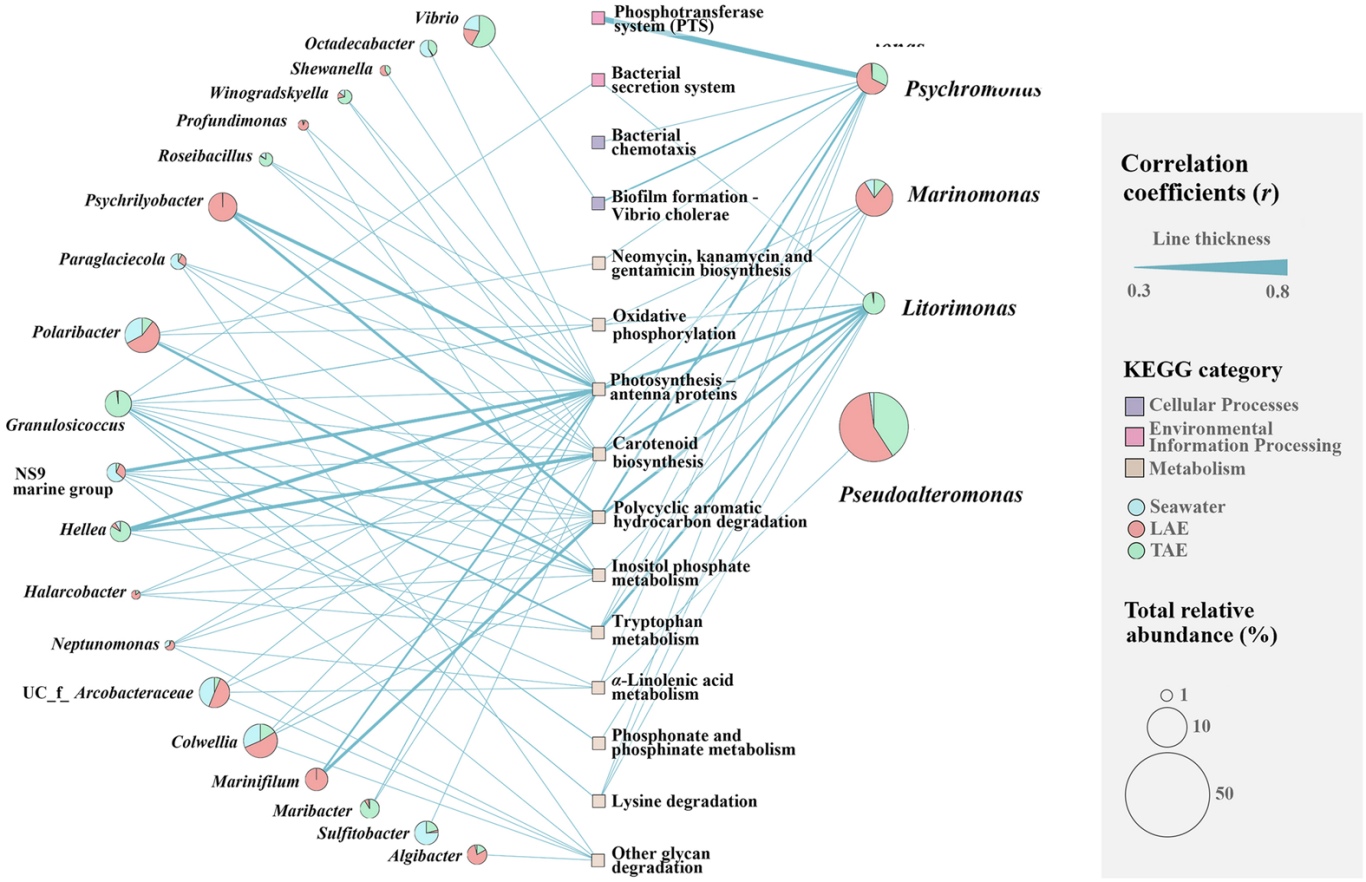
Spearman’s rank-order correlation networks indicate remarkable positive relationships (*r* > 0.3, *p* < 0.05) between bacterial taxa and KEGG functional genes at the tertiary level. Edge thickness reflects the correlation strength. Pie charts represent the total mean relative abundances of the bacterial taxa across seawater, LAE, and TAE, with segments indicating their distribution within each environment

To identify the bacterial taxa linked to the metabolic differences observed among seawater, LAE, and TAE, we performed a Spearman rank-order correlation analysis between bacterial abundances and their tertiary-level KEGG functional genes, as predicted by PICRUSt2 (Fig. 7). The results revealed that *Psychromonas*, which was more abundant in LAE, exhibited remarkable positive correlations with “phosphotransferase system (PTS),” “biofilm formation-*Vibrio cholerae*,” “bacterial chemotaxis,” “*α*-linolenic acid metabolism,” and “neomycin, kanamycin, and gentamicin biosynthesis.” *Marinomonas* was markedly associated with “photosynthesis-antenna proteins” and “carotenoid biosynthesis.” Notably, *Litorimonas* was linked to multiple KEGG functional categories, including “bacterial secretion system,” “photosynthesis-antenna proteins,” “oxidative phosphorylation,” and “carotenoid biosynthesis,” which may contribute to the interactions with marine algae. In addition to these core taxa, other bacteria with more abundance in LAE and/or TAE, including *Psychrilyobacter*, *Granulosicoccus*, *Hellea*, *Marinifilum*, and *Vibrio*, showed significant positive correlations with metabolic functions relevant to marine algal interactions, such as “biofilm formation-*Vibrio cholerae*,” “bacterial secretion system,” “photosynthesis-antenna proteins,” “oxidative phosphorylation,” and “carotenoid biosynthesis.” Overall, these findings suggest that the LAE and TAE bacterial communities, distinct from those in seawater, may play vital roles in metabolic interactions within the phycosphere of marine algae. However, *Pseudoalteromonas*, despite being abundant in LAE and TAE, exhibited only a weak positive correlation with “*α*-linolenic acid metabolism.”

### Isolation and genome sequencing of potential core taxa from the phycospheres and their phylogenetic inference

A diverse range of bacterial strains was successfully isolated from the phycosphere of marine macroalgae (Table S5), including strains from the genera *Pseudoalteromonas*, *Psychromonas*, *Marinomonas*, and *Litorimonas*, which were identified as potential core taxa. The most abundant strains within these core taxa, determined by comparing the 16S rRNA gene amplicon patterns, were selected as representatives (Table S6). Their complete genomes were sequenced to investigate any potential metabolic interactions with marine macroalgae. The comparison revealed that isolates RW-H-Ap-1, GE-S-Ul-11, RS-M-Aa-14, and RW-G-Af-16 were closely related to the type strains of *Pseudoalteromonas atlantica, Psychromonas arctica, Marinomonas algicola,* and *Litorimonas taeanensis*, respectively, with sequence similarities ranging from 96.8 to 99.9%.

Phylogenetic analysis based on 120 single-copy marker proteins confirmed these classifications, with the isolates forming distinct lineages within their respective genera (Fig. S6). However, the ANI and dDDH values between these isolates and their closest type strains were below the standard thresholds for defining prokaryotic species (ANI: ~ 95%; dDDH: 70%), suggesting that these may represent novel species within the respective genera (Riesco and Trujillo 2024). To further investigate the metabolic interactions of the bacteria with marine algae and adaptations to LAE and/or TAE, their metabolic traits were comprehensively analyzed using bioinformatics and genomic data.

### Metabolic features of the RW-H-Ap-1, GE-S-Ul-11, RS-M-Aa-14, and RW-G-Af-16 strains

#### Metabolic features of RW-H-Ap-1

RW-H-Ap-1, a *Pseudoalteromonas* species, contains two chromosomes (3439 and 746 kb long) and encodes complete pathways for glycolysis, gluconeogenesis, the pentose phosphate (PP) pathway, the tricarboxylic acid (TCA) cycle, and oxidative phosphorylation, indicating its capacity for aerobic respiration. It features a wide array of CAZymes, including 29 glycoside hydrolases (GHs) such as GH3, GH13, GH16, and GH23; 17 glycosyltransferases (GTs); and three carbohydrate esterases (Table S7). Additionally, it possesses genes for synthesizing B vitamins, such as thiamin (B_1_), niacin (B_3_), pantothenate (B_5_), pyridoxine (B_6_), biotin (B_7_), and folate (B_9_), along with genes for producing 2-hydroxy-phenylacetate (2-OH-PAA) from phenylalanine (Fig. S7). Genes encoding desferrioxamine (ACJ3W8_17260–17415) and those involved in converting arginine to agmatine (*speA*; ACJ3W8_10025, 14690), agmatine to putrescine (*speB*; ACJ3W8_10020), and putrescine to spermidine (*speE*; ACJ3W8_14695) were also identified. It also features a chemotaxis-associated gene cluster, *cheVAWY* (ACJ3W8_00125, 14830, 11340, 11345, 11370, 14825, and 11380), and *cheC* (ACJ3W8_17900), as well as genes encoding three ribosomally synthesized and post-translationally modified peptides (RiPP)-like compounds (ACJ3W8_02260–02305, 09250–09300, and 16105–16130).

#### Metabolic features of GE-S-Ul-11

GE-S-Ul-11, a *Psychromonas* species, harbors a 3915 kb long chromosome which encodes diverse transport systems for organic molecules, phosphates, amines, amino acids, and metals, reflecting its metabolic versatility. It demonstrates complete pathways for glycolysis, gluconeogenesis, PP metabolism, and the TCA cycle, facilitating aerobic respiration. Its genome encodes various CAZymes, including 18 GHs, 26 GTs (e.g., GT4, GH32, GH13, and GT5), and 14 polysaccharide lyases (PLs), with eight PL7s associated with alginate degradation and one PL6 encoding rhamnogalacturonan lyase (Table S7). Its chromosome also contains two auxiliary activity (AA) genes, including AA3 encoding glucose-methanol-choline oxidoreductases. It has genes for synthesizing all B vitamins, including B_1_, riboflavin (B_2_), B_3_, B_5_, B_6_, B_7_, B_9_, and cobalamin (B_12_). Additionally, it possesses genes for converting arginine to agmatine (*speA*; ACJ3XG_13370), agmatine to putrescine (*speB*; ACJ3XG_05640, 13375), and putrescine to spermidine (*speE*; ACJ3XG_00490, 14900), as well as for synthesizing phenylacetic acid (PAA) from phenylalanine (Fig. S7). Bioinformatics revealed that the strain harbors a nonribosomal peptide synthetase (*NRPS*) gene cluster (ACJ3XG_16410–16595) and the *entABCDEF* gene cluster (ACJ3XG_16480, 16495–16515), involved in the synthesis of enterobactin, a siderophore.

#### Metabolic features of RS-M-Aa-14

RS-M-Aa-14, a *Marinomonas* species, has a 4581 kb long chromosome that encodes transport systems for sugars, sugar alcohols, amines, amino acids, and metals. It exhibits complete pathways for glycolysis, gluconeogenesis, the PP pathway, and the TCA cycle, supporting aerobic respiration. The chromosome encodes 28 GHs and 21 GTs, including GH42 and GH73 for *β*-galactosidase, enabling the metabolism of diverse algal polysaccharides. It also contains genes for synthesizing all B vitamins except B_3_; genes for converting arginine to agmatine (*speA*; ACJ3XJ_02340) and agmatine to putrescine (*aguA* and *aguB*; ACJ3XJ_01140, 02345); and genes for producing PAA from phenylalanine (Fig. S7). It also harbors *bfr* (ACJ3XJ_02365, 02370) and the *entABCEF* cluster (ACJ3XJ_08190–08220) involved in the production of the siderophores bacterioferritin and enterobactin, respectively. It also harbors *dmdBCD*s for dimethylsulfoniopropionate (DMSP) metabolism, though it lacks *dmdA*, required to convert DMSP to 3-methylmercaptopropionate (MMPA) (Fig. S8). Additionally, the genome contains *luxS* (ACJ3XJ_21275) for quorum sensing and *vgrG* (ACJ3XJ_20745) and *hcp* (ACJ3XJ_20740), key components of the Type VI secretion system. It also harbors an NRPS operon (ACJ3XJ_08095–08320), two RiPP-like clusters (ACJ3XJ_09920–09955, 17140–17200), and a biosynthesis operon for ectoine (ACJ3XJ_20625–20660).

#### Metabolic features of RW-G-Af-16

RW-G-Af-16, a *Litorimonas* species, possesses a 2548 kb long chromosome. It possesses complete pathways for glycolysis, gluconeogenesis, the PP pathway, the TCA cycle, and oxidative phosphorylation, supporting aerobic respiration. The chromosome encodes 18 GHs, 10 GTs, and 5 PLs, including PL6 and PL17 for alginase and PL7 for carrageenase (Table S7). It contains genes for synthesizing vitamins B_1_, B_5_, and B_9_ and converting agmatine to putrescine (*speB*; ACJ3XI_07265) and phenylalanine to 2-OH-PAA (Fig. S7). It also harbors a complete *dmdABC* and *acuH* gene set for metabolizing DMSP into methanethiol, carbon dioxide, and acetaldehyde via the demethylation pathway (Fig. S8). Additionally, it contains a terpene biosynthesis cluster (ACJ3XI_04230–04460) for synthesizing zeaxanthin from *β*-carotene (Fig. S9), as well as a nitrogen fixation-associated gene cluster (*fixNOQPGHIS*) for producing ammonia from nitrogen (Fig. S10).

## Discussion

Symbiotic metabolic interactions between heterotrophic bacteria and marine phototrophs, such as corals, algae, diatoms, dinoflagellates, and cyanobacteria, are well-documented; they significantly influence the physiology, metabolism, survival, and growth of both partners (Amin et al. 2015; Cirri and Pohnert 2019; Egan et al. 2013). Numerous vital metabolic interactions between symbiotic heterotrophic bacteria and host phototrophs, particularly corals, diatoms, and microalgae, which are crucial for their growth and survival, have been identified (Amin et al. 2015; Barak-Gavish et al. 2023; Cooper et al. 2019; Rambo et al. 2020; Santoro et al. 2021). For example, a *Sulfitobacter* species promotes diatom growth by secreting indole-3-acetic acid, utilizing diatom-derived tryptophan (Amin et al. 2015). Bacteria-produced vitamin B_12_ is also essential for the growth of photoautotrophs (Cooper et al. 2019; Croft et al. 2005). *Emiliania huxleyi* produces benzoic acid, which supports its coexistence with *Sulfitobacter* D7 (Barak-Gavish et al. 2023). However, our understanding of these metabolic interactions is still limited, as most studies have focused on only a few species.

Several studies have investigated bacterial communities associated with marine algae to better understand the macroalgae–bacteria interactions (Bondoso et al. 2017; Burgunter-Delamare et al. 2023; Ihua et al. 2020; King et al. 2023; Saha et al. 2024; Wichard 2023). However, many of these are limited to certain macroalgal species, such as the green macroalgal genus *Ulva* (Califano et al. 2020; Spoerner et al. 2012; Wichard 2023). As macroalgae–bacteria interactions may be specific to individual macroalgal species, comprehensively understanding common functional interactions across diverse marine algae remains challenging. To gain deeper insights, it is essential to identify the core bacterial species associated with marine phototrophs and investigate their relationship, an issue being addressed by recent research (Astudillo-García et al. 2017; Kim et al. 2024; Lu et al. 2023; Park et al. 2022). These interactions are primarily driven by the exchange of nutrients and small molecules within the phycosphere—a microenvironment rich in host and bacterial metabolites, distinct from the surrounding seawater (Califano et al. 2020; Seymour et al. 2017; Cirri and Pohnert 2019). While marine macroalgae are gaining attention for their ecological and industrial significance (de Barros Medeiros et al. 2022), their interactions with heterotrophic bacteria remain much less understood compared to other marine phototrophs.

This study analyzed the bacterial communities within the phycosphere of 43 marine macroalgal species collected from three regions, along with those residing in the surrounding seawater. The phycospheric microbial communities differed remarkably in composition from those in the seawater (Burke et al. 2011; Califano et al. 2020), suggesting that the phycosphere provides environmental conditions that vary from those of the surrounding seawater. The 16S rRNA gene-based analysis in this study further confirmed that the bacterial communities in the phycosphere (LAE and TAE) were distinct from those in seawater (Figs. 3 and 4C).

Characterizing microbial communities within the phycosphere of marine algae is challenging due to their complex, dynamic, and variable nature. These communities are shaped by biotic factors such as algal phylogeny (or color), growth stage, and health status, as well as environmental factors including temperature, pH, dissolved oxygen, nutrient availability, salinity, and depth (Hengst et al. 2010; Lemay et al. 2021; Lu et al. 2023; Marzinelli et al. 2015; Wood et al. 2022). Macroalgal phylogeny (color) has been identified as a key determinant of bacterial community composition within the phycosphere (Bondoso et al. 2017; Lachnit et al. 2009; Nahor et al. 2024), with evidence suggesting that phylogeny plays a more remarkable role than habitat conditions (Lachnit et al. 2009). Additionally, certain bacterial taxa appear to be species-specific rather than broadly distributed among different macroalgae (Ihua et al. 2020).

However, our findings indicate that the bacterial communities within the phycosphere of the marine macroalgae may be influenced more by the collection site than by algal phylogeny (or color) (Figs. 3, S3, S4, and S5). This observation indicates that site-specific environmental conditions may have a greater impact on bacterial composition than variations in polysaccharide composition among the red, brown, and green algae. These results contrast with those of previous studies where bacterial communities were primarily structured by algal phylogeny rather than geographic variations (Chen et al. 2022; Lachnit et al. 2009; Nahor et al. 2024). The variations observed suggest that physicochemical factors such as temperature, salinity, and nutrient availability may exert a more robust influence than host phylogeny alone (Martínez et al. 2012; Pei et al. 2021; Roth‐Schulze et al. 2016; Stratil et al. 2014). The lack of any significant differentiation based on macroalgal phylogeny in our study may be due to the presence of shared core microbial taxa across various algal hosts, as reported by studies on epiphytic microbiomes (Lu et al. 2023; Lemay et al. 2018). Additionally, our results align with previous findings that macroalgae-associated microbiomes are shaped by geographic and habitat conditions (Wood et al. 2022). In addition, the same bacterial species or microbiome can exhibit different ecophysiological functions in response to changing environmental conditions, as reported by Seyedsayamdost et al. (2011) and Hmani et al. (2024). These findings underscore the complexity of microbial community assembly within the macroalgal phycosphere and highlight the need for further research into site-specific environmental factors that influence bacterial diversity and function.

Bacterial communities in seawater, LAE, and TAE—particularly between LAE and TAE—exhibited greater differentiation (Fig. 3C) than those associated with algal phylogeny (Fig. 3A) or collection site (Fig. 3B). This result indicates that the environmental conditions of the seawater, LAE, and TAE are more distinct from each other and are more influential than collection site or algal type. Comparing bacterial communities across these environments may help identify potential symbiotic species associated with marine macroalgae. We identified plausible core taxa that most likely interact with marine algae through IndVal analysis, which examined the differences among LAE, TAE, and seawater bacterial communities, as well as their frequencies in LAE and TAE (Fig. 4). It identified *Pseudoalteromonas*, *Psychromonas*, *Marinomonas*, and *Litorimonas* as the core taxa that can help distinguish LAE and/or TAE communities from the seawater ones, suggesting their potential roles in interactions with marine macroalgae. Specifically, *Pseudoalteromonas* emerged as a common core taxon for LAE and TAE. Meanwhile, *Psychromonas* and *Marinomonas* were the core taxa in LAE, and *Litorimonas* in TAE, indicating that they may have distinct physiological and phenotypic traits that facilitate their interactions with algal hosts. These taxa are abundant in the rhizosphere and phycosphere of marine algae (Chen et al. 2020; Ihua et al. 2020; Paix et al. 2021; Qu et al. 2021; Selvarajan et al. 2019), further supporting their potential roles as core taxa involved in metabolic interactions with marine algae. Co-occurrence network analysis revealed that *Pseudoalteromonas*, *Psychromonas*, *Marinomonas*, and *Litorimonas* showed remarkable positive or negative correlations with numerous other taxa, forming central nodes within the network (Fig. 5). “Keystone taxa” or “hubs” are known to play critical roles in ecological networks by mediating interactions with multiple microbial taxa (Banerjee et al. 2018; Meng et al. 2023). Therefore, these bacterial taxa may act as “potential keystone taxa,” shaping the structure of ecological communities through various symbiotic or antagonistic interactions within the phycosphere.

Here, we isolated bacterial strains from the potential keystone taxa *Pseudoalteromonas*, *Psychromonas*, *Marinomonas*, and *Litorimonas*, which were metabolically associated with marine macroalgae, and sequenced their complete genomes. Genome-based taxonomic analyses, including ANI and dDDH values, revealed that all isolates represented new species within the respective genera. This finding suggests that they may possess unique metabolic traits critical for their roles as keystone species during symbiosis with marine algae or for survival within the phycosphere. To further investigate these interactions, we conducted bioinformatic analyses to explore their metabolic traits related to interactions with marine macroalgae.

Strain RW-H-Ap-1, identified as a core species of *Pseudoalteromonas* in both LAE and TAE (Fig. 4A, B), possesses a diverse array of CAZyme-encoding genes (Table S7), indicating its capability to degrade polysaccharides from various marine algae. This strain can synthesize several B vitamins (B_1_, B_3_, B_5_, B_6_, B_7_, and B_9_), potentially supplying essential vitamins to marine algae or associated microbiota that cannot synthesize them (Cooper et al. 2019). Additionally, RW-H-Ap-1 may produce 2-OH-PAA, a compound that promotes algal growth and enhances oxidative stress resistance (Fries 1977; Kim et al. 2024) (Fig. S7). It is also capable of synthesizing polyamines such as putrescine and spermidine, which help protect algal cells from abiotic stress (Xu et al. 2021). Its genome contains the *cheVAWY* chemotaxis-associated gene cluster, which is also present in closely related *Pseudoalteromonas* species (Fig. S6A). An additional *cheC* was also identified; it was not found in the related species, suggesting an enhanced chemotactic response and adhesion compared to other *Pseudoalteromonas* species. Furthermore, RW-H-Ap-1 may synthesize three RiPP-like compounds that influence microbial community dynamics and produce desferrioxamine, a siderophore that enhances iron bioavailability for marine macroalgae. These diverse metabolic traits are likely essential for the survival of RW-H-Ap-1 and its symbiotic role as a core taxon within the LAE and TAE of various marine macroalgae.

Strain GE-S-Ul-11, identified as a core species of *Psychromonas* in LAE (Fig. 4A), also possesses a wide range of CAZyme-encoding genes (Table S7), including eight *PL7*s for alginate degradation, a major component of green algal cell walls, as well as *PL6* and *AA3* for metabolizing complex carbohydrates in green algae (Sun et al. 2023; Xia et al. 2023). These features indicate that GE-S-Ul-11 is specialized in degrading polysaccharides from green algae (Table S7). It can synthesize all B vitamins, a trait also observed in a closely related *Psychromonas* species (Fig. S6B), suggesting that it may play a crucial role in supplying B vitamins to marine algae or symbiotic microbiota, thus contributing to ecosystem sustainability. Additionally, GE-S-Ul-11 may produce PAA (Fig. S7), known to promote algal growth and stress resistance (Fries 1977), as well as the production of polyamines such as putrescine and spermidine. The strain also harbors genes for synthesizing an NRPS compound, while closely related *Psychromonas* strains produce RiPP-like compounds (analyzed in this study), indicating that GE-S-Ul-11 may produce distinct antimicrobial compounds. Furthermore, it has the potential to synthesize enterobactin, a high-affinity siderophore that enhances iron uptake (Qi and Han 2018). In contrast, *P*. *aquatilis* TI.1.05, isolated from seawater, lacks these genes, and *P*. *arctica* DSM 14288^ T^, also a seawater isolate, contains only *entC* (analyzed in this study). This observation suggests that GE-S-Ul-11, isolated from marine algae, may markedly enhance iron availability for marine macroalgae through enterobactin production.

Strain RS-M-Aa-14, identified as a core species of *Marinomonas* in LAE (Fig. 4A), possesses a diverse set of CAZyme-encoding genes (Table S7), including GH42 and GH73 (*β*-galactosidase), indicating its capability to degrade cell wall components of various marine algae, particularly red algae. This strain may synthesize all B vitamins except B_3_ and produce agmatine, putrescine, and 2-phenylacetamide (Fig. S7). Similar to other closely related *Marinomonas* species (Fig. S6C), RS-M-Aa-14 may produce bacterioferritin, suggesting that this trait is common to the genus. Notably, only RS-M-Aa-14 and *M*. *rhizomae* IVIA-Po-145^ T^, which was isolated from seagrass, appear to be capable of synthesizing enterobactin (analyzed in this study), indicating that siderophore production may be crucial for survival within the phycosphere of marine algae.

DMSP, the most abundant organo-sulfur compound in the ocean, plays a critical role not only in the global carbon and sulfur cycles but also functions as an osmoprotectant and a general stress-mitigating compound, helping cells withstand environmental stressors such as salinity and oxidative stress. It also serves as a vital source of sulfur and energy for various marine organisms (Carrión et al. 2023). In *Ulva*, DMSP is a potent infochemical within the algal phycosphere, functioning as a signaling molecule that attracts specific bacterial taxa (Kessler et al. 2018). As a result, the biosynthetic and degradation pathways of DMSP, along with its ecological roles in algal–bacterial interactions, have been extensively studied. Although DMSP is predominantly synthesized by marine algae, recent studies have demonstrated that substantial amounts are also produced by marine bacteria (Carrión et al. 2023; Curson et al. 2017). In marine ecosystems, DMSP produced by algae and bacteria is primarily metabolized into methanethiol, carbon dioxide, and acetaldehyde through the demethylation pathway, which involves four key enzymes: *DmdA*, *DmdB*, *DmdC*, and *DmdD*/*AcuH* (Fig. S8) (Wang et al. 2022). RS-M-Aa-14 contains *dmdBCD*s but lacks *dmdA*, which differentiates it from other closely related *Marinomonas* strains that lack both *dmdAB*s (Fig. S8B). This genetic configuration provides RS-M-Aa-14 with an enhanced capacity for DMSP metabolism compared to other strains, although cooperation with other microbes may be necessary for complete DMSP metabolism. Additionally, RS-M-Aa-14 also harbors *luxS*, involved in quorum sensing as well as *vgrG* and *hcp* associated with the Type VI secretion system (Cianfanelli et al. 2016), which are absent in related *Marinomonas* species (Fig. S6C). These genes may facilitate interactions between symbiotic bacteria and marine algae and are most likely crucial for the competitive survival of RS-M-Aa-14 within the marine phycosphere (Boyer et al. 2009). Furthermore, RS-M-Aa-14 may synthesize NRPS and RiPP compounds, as well as ectoine, a compatible solute that enhances the resilience of the host algae to environmental stresses (Fenizia et al. 2020).

Strain RW-G-Af-16, identified as a core species of the genus *Litorimonas* in TAE (Fig. 4B), harbors a broad array of CAZyme-encoding genes (Table S7), enabling it to metabolize various algal polysaccharides. This strain synthesizes a limited range of B vitamins (B_1_, B_5_, and B_9_), indicating a reliance on other commensal organisms for other vitamins. It may also produce putrescine and 2-OH-PAA, similar to other potentially core taxa (Fig. S7). RW-G-Af-16 possesses a complete set of DMSP metabolism-related genes, *dmdABC* and *acuH* (Fig. S8), unlike closely related *Litorimonas* strains that lack the DMSP demethylase gene, *dmdA* (Fig. S8C). This finding suggests that RW-G-Af-16 may contribute more effectively to the sulfur cycle within the phycosphere. Notably, it may synthesize zeaxanthin, a compound that helps coral holobionts mitigate oxidative stress under high thermal and light conditions (Motone et al. 2020) (Fig. S9A). In contrast, closely related strains such as *L*. *taeanensis* DSM 22008^ T^ and *L*. *cladophora* KCTC 23968^ T^ lack *crtZ*, which encodes the enzyme required for the conversion of *β*-carotene to zeaxanthin (Fig. S9B). Unlike *β*-carotene, zeaxanthin can bind to the retinal portion of rhodopsin, enhancing light energy transfer (Chazan et al. 2023). This observation indicates that RW-G-Af-16 may reduce oxidative stress and improve light energy utilization in algal hosts more effectively than other *Litorimonas* strains. Additionally, it may fix nitrogen to ammonia (Fig. S10), potentially promoting algal growth (Kim et al. 2024).

**Fig. 8:**
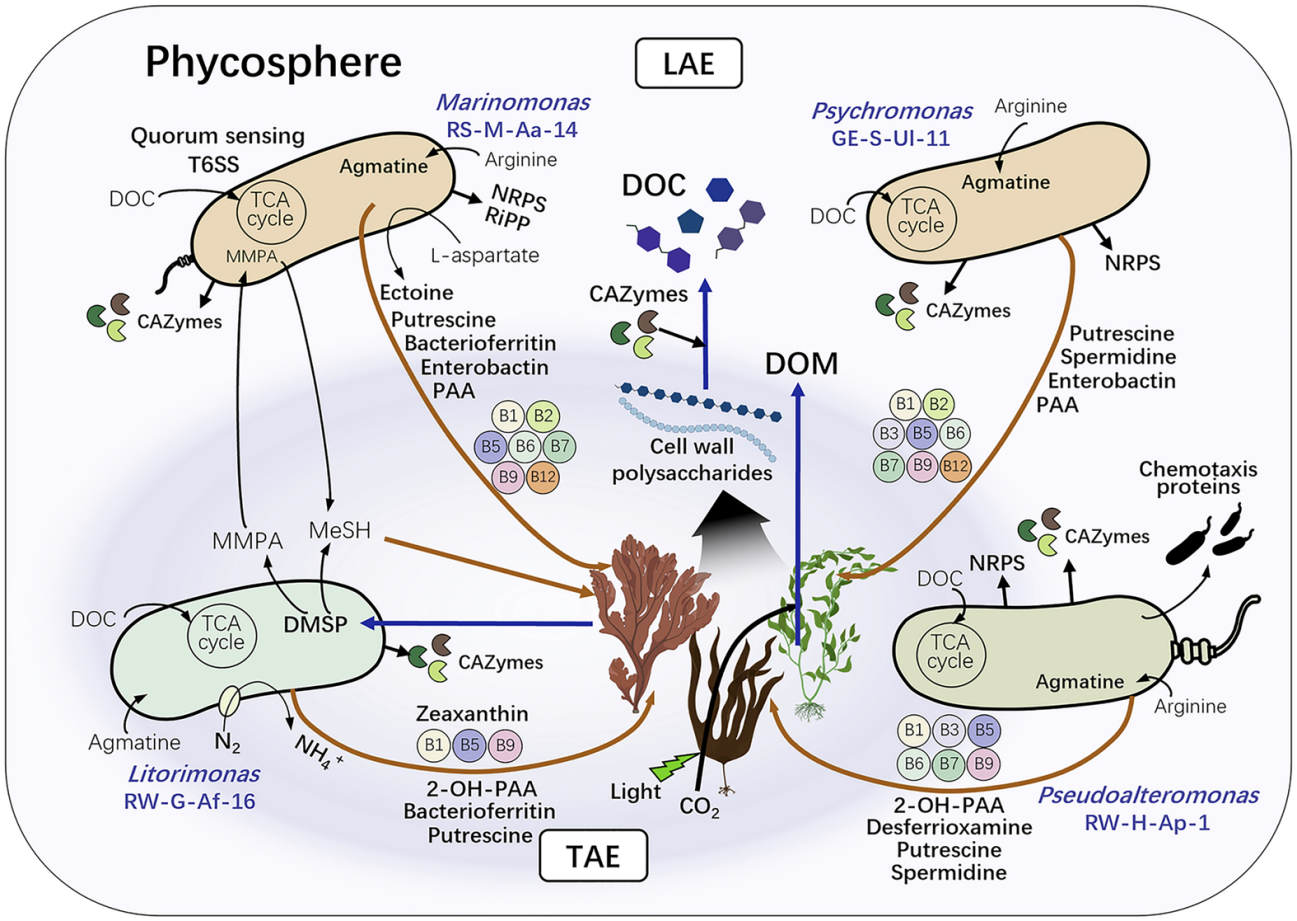
A schematic illustration of the predicted metabolic traits of the four potentially core species involved in interactions with marine macroalgae within the phycosphere, and identified based on genomics. DMSP, dimethylsulfoniopropionate; MMPA, 3‐methylmercaptopropionate; MeSH, methanethiol; PAA, phenylacetate; 2-OH-PAA, 2-hydroxy-phenylacetate; DOC, dissolved organic carbon; DOM, dissolved organic matter; and T6SS, Type VI secretion system

The metabolic traits of four potential keystone species interacting with marine macroalgae identified by bioinformatic analyses are schematically illustrated (Fig. 8). These core species exhibit distinct metabolic traits within the phycosphere of marine algae. For instance, *Litorimonas* RW-G-Af-16 is the only species capable of fixing nitrogen, thereby promoting the growth of marine algae and associated microbiota. In contrast, only *Marinomonas* RS-M-Aa-14 and *Psychromonas* GE-S-Ul-11 can synthesize vitamin B_12_, an essential nutrient for biological metabolism. The DMSP utilization pathway is restricted to *Litorimonas* RW-G-Af-16 and *Marinomonas* RS-M-Aa-14. The diversity of CAZymes and siderophores produced by these core species indicates their complementary roles in supporting algal growth. Additionally, they produce antimicrobials that help maintain ecosystem stability by preventing pathogen invasion and regulating competition within the phycosphere. Figure 8 highlights that no single species possesses all the metabolic traits necessary for ecosystem stability and a completely viable symbiotic relationship with marine algae. Instead, each species contributes unique metabolic capabilities that collectively foster a balanced ecosystem. Notably, all four keystone strains are associated with the production of PAA or 2-OH-PAA, compounds that promote algal growth and reduce oxidative stress; however, none produces both simultaneously. While our characterization of the core species provides valuable insights into metabolic interactions within the phycosphere, other microbiota, considered less critical, are also most likely essential for maintaining ecosystem stability and facilitating interactions with marine algae.

Despite the valuable findings presented in this study, several limitations should be acknowledged. First, the metabolic functions of microbial communities were predicted using PICRUSt2 analysis based on 16S rRNA gene sequences rather than metagenomic data. As a result, the inferred functional profiles may not fully reflect the actual metabolic landscape of the phycospheric microbiota due to the inherent constraints of predictive tools that rely on the availability of a reference genome. Second, while potential metabolic interactions between macroalgae and core bacterial taxa identified through IndVal analysis were inferred using genome-based bioinformatic approaches, these remain only theoretical without any experimental validation. Thus, the predicted metabolic traits and symbiotic roles should be considered hypothetical until confirmed by direct experimental evidence. To address these limitations, future studies should aim to validate the predicted gene functions through laboratory-based approaches such as co-culture experiments, gene expression analyses, and other functional assays involving isolated representative strains. Furthermore, to gain a broader understanding of the metabolic interactions within the phycosphere, research should extend beyond the core taxa to include the wider microbial community. Functional roles of these microbiota should be further explored by verifying the symbiosis-related traits and applying integrated metaomic approaches—such as metagenomics, metatranscriptomics, metaproteomics, and metabolomics—to assess microbial activity under natural marine conditions. Additionally, since environmental factors such as temperature, light availability, and algal physiological status vary seasonally, bacterial community structures are also most likely influenced by seasonality. However, our study was based on single-time-point sampling conducted in April across different coastal regions. Future research should incorporate seasonal samplings to better capture temporal dynamics and evaluate seasonal impacts on the phycospheric microbiota. Such efforts can substantially strengthen the conclusions of this study and enhance our understanding of macroalgae–bacteria interactions.

## Conclusion

Symbiotic metabolic interactions between heterotrophic bacteria and marine macroalgae are essential for their growth and survival. However, the specific core bacteria associated with marine macroalgae and the metabolic interactions between the two remain poorly understood. In this study, we statistically compared the bacterial communities in the surrounding seawater as well as the LAE and TAE of 43 marine macroalgal species. We identified *Pseudoalteromonas*, *Psychromonas*, *Marinomonas*, and *Litorimonas* as potentially core (keystone) taxa that may form symbiosis with marine macroalgae. By isolating the bacterial strains belonging to these core taxa and characterizing their metabolic traits related to their interactions with marine macroalgae through bioinformatics and genomics, this study contributes to a broader understanding of the complex metabolic interactions between heterotrophic bacteria and marine macroalgae within the phycosphere.

## Supplementary Information

Below is the link to the electronic supplementary material.

Supplementary file1 (DOCX 6528 KB)

## Data Availability

The bacterial 16S rRNA gene sequencing Illumina data derived from this study are publicly available in the NCBI Short Read Archive under accession number PRJNA1107946. The whole genome sequences of strains RW-H-Ap-1, GE-S-Ul-11, RS-M-Aa-14, and RW-G-Af-16 have been deposited in GenBank under the accession numbers CP176466–7, CP176468, CP176470, and CP176469, respectively.

## Declarations

### Animal and human right statement

This article does not contain any studies involving human participants or animals conducted by any of the authors.
