# Supplementary material for: Identifying potential keystone bacterial species within the phycosphere of marine algae and unveiling their metabolic characteristics
Source: Mar Life Sci Technol. 2025 Oct 28;7(4):989–1007. doi: 10.1007/s42995-025-00325-6 (PMC12662954; doi:10.1007/s42995-025-00325-6)
Supplement: Supplementary file 1 — Supplementary file1 (DOCX 6528 KB) [file 42995_2025_325_MOESM1_ESM.docx]

**Supplementary materials**

**Fig. S1** Geographic locations of the collection sites of marine macroalgae used in this study along the Korean Peninsula coast. Red dots indicate the specific collection sites, and seawater temperatures at these locations are provided in parentheses.


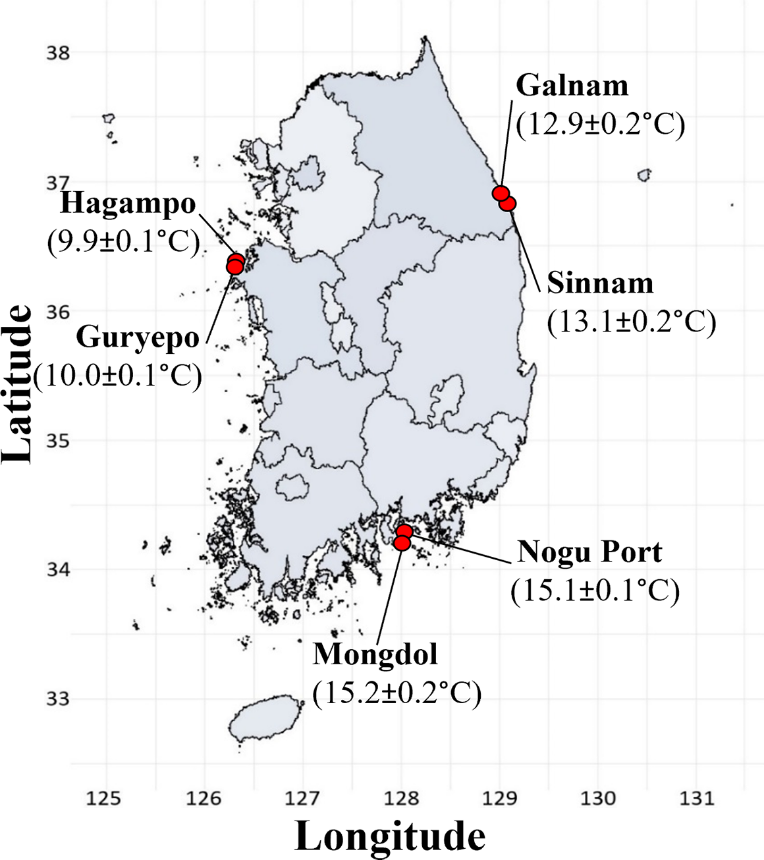


**Fig. S2** Neighbor-joining phylogenetic tree of marine macroalgae collected from the West Sea, South Sea, and East Sea in South Korea, based on their ribulose-1,5-bisphosphate carboxylase gene (*rbcL*) sequences (**A**), and clustering dendrograms, based on the Bray-Curtis dissimilarity metric, of bacterial communities at the genus level derived from loosely attached environments (LAE, **B**) and tightly attached environments (TAE, **C**) of marine macroalgae. In panel A, the *rbcL* sequence of *Euglena viridis* (U21010) was used as the outgroup, and in panels B and C, the bacterial community of seawater samples served as the outgroup. The phylogenetic tree and dendrograms were constructed and visualized using MEGA11.


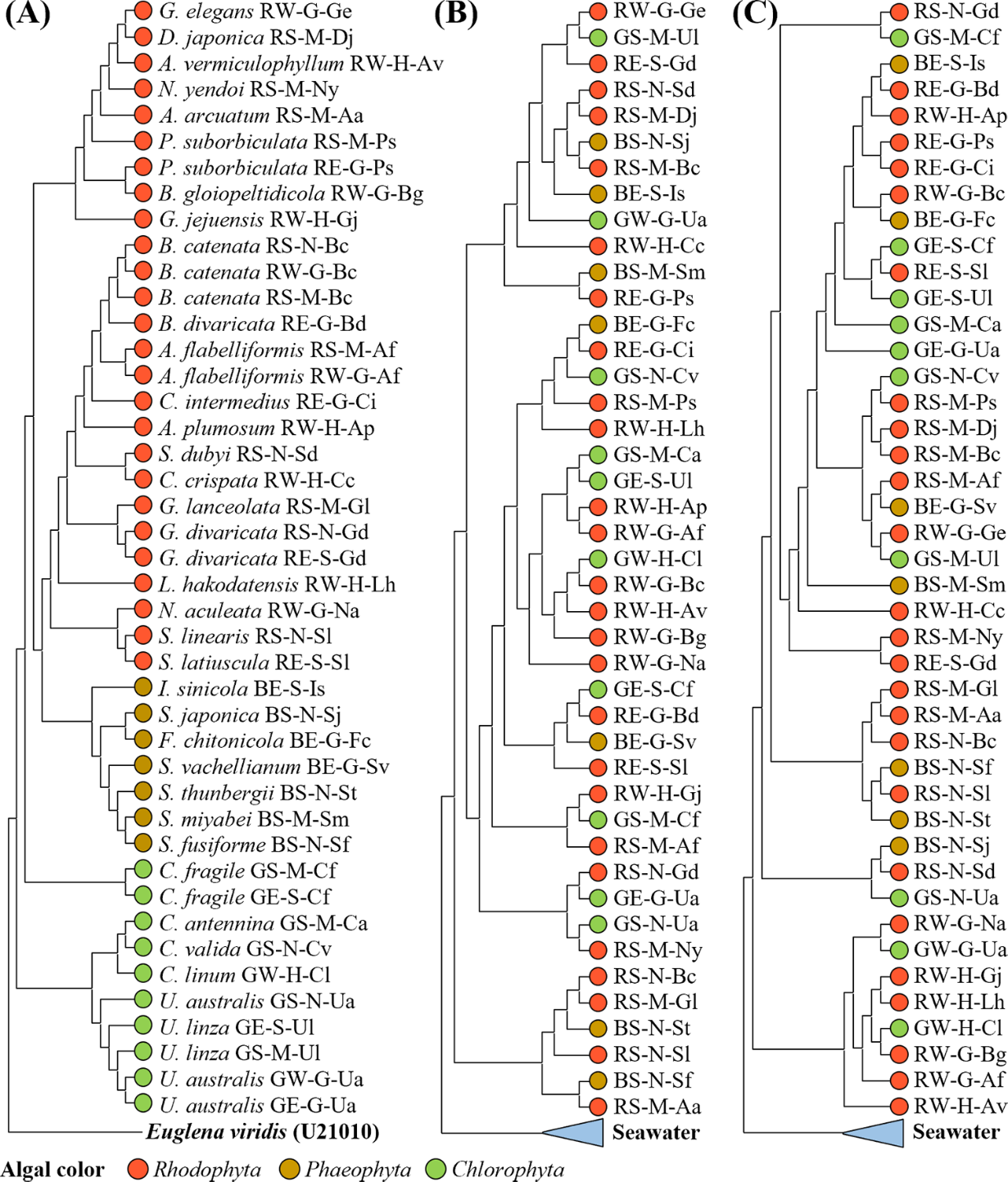


**Fig. S3** Box plots showing the relative abundances of major core taxa shared across algal colors (red, brown, and green) within LAE (A) and TAE (B). Statistical differences were assessed using the Wilcoxon rank-sum test, and no significant differences in relative abundances were observed among the groups in either region.**
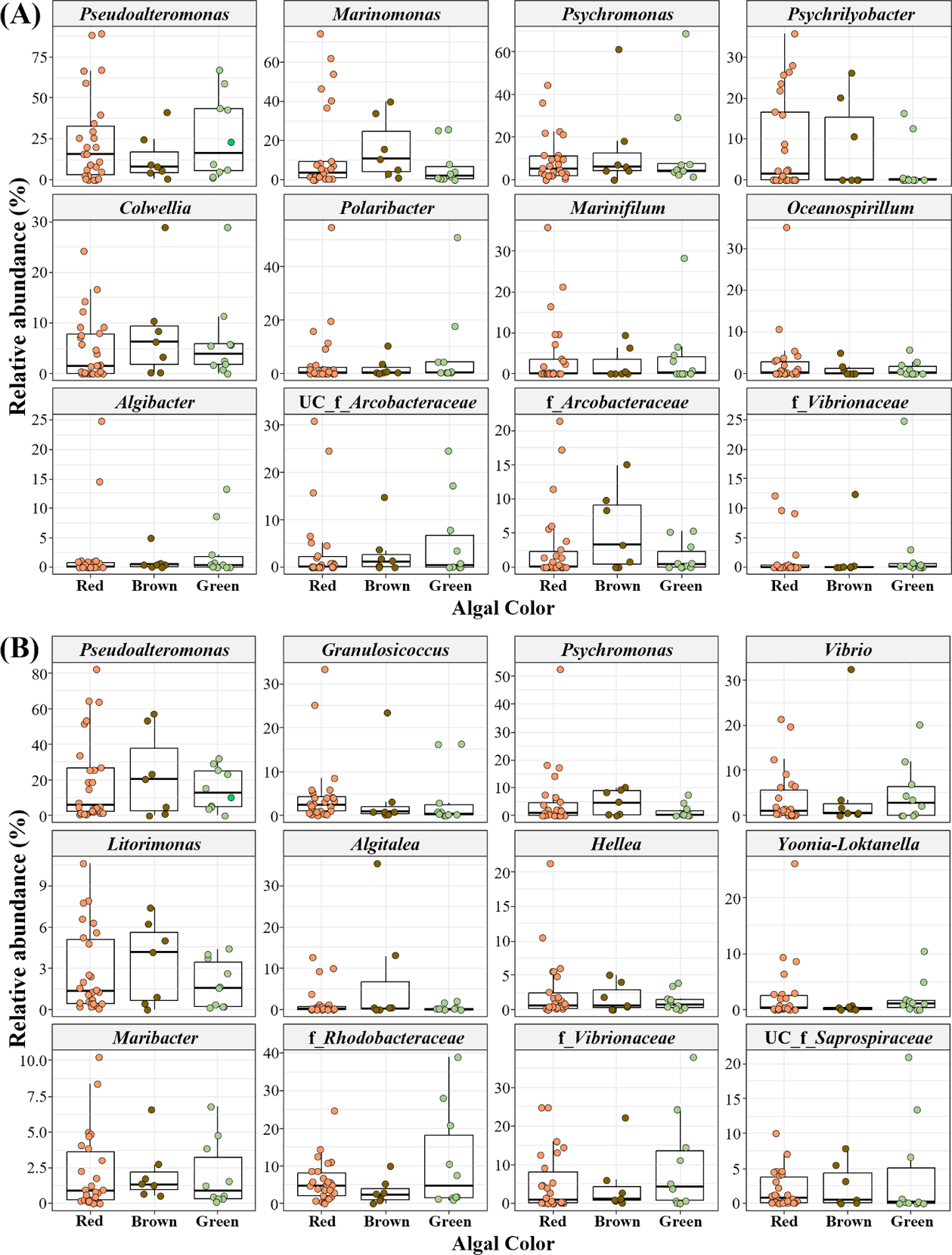
**

**Fig. S4** Non-metric multidimensional scaling (NMDS) plots comparing bacterial communities in the loosely attached environment (LAE) and tightly attached environment (TAE) of red, brown, and green macroalgae collected from the East (A), West (B), and South (C) Sea. Bacterial community dissimilarities based on algal color were assessed using the Bray-Curtis method, with statistical significance evaluated by PERMANOVA. No significant differences were observed among the groups (*p* > 0.2).


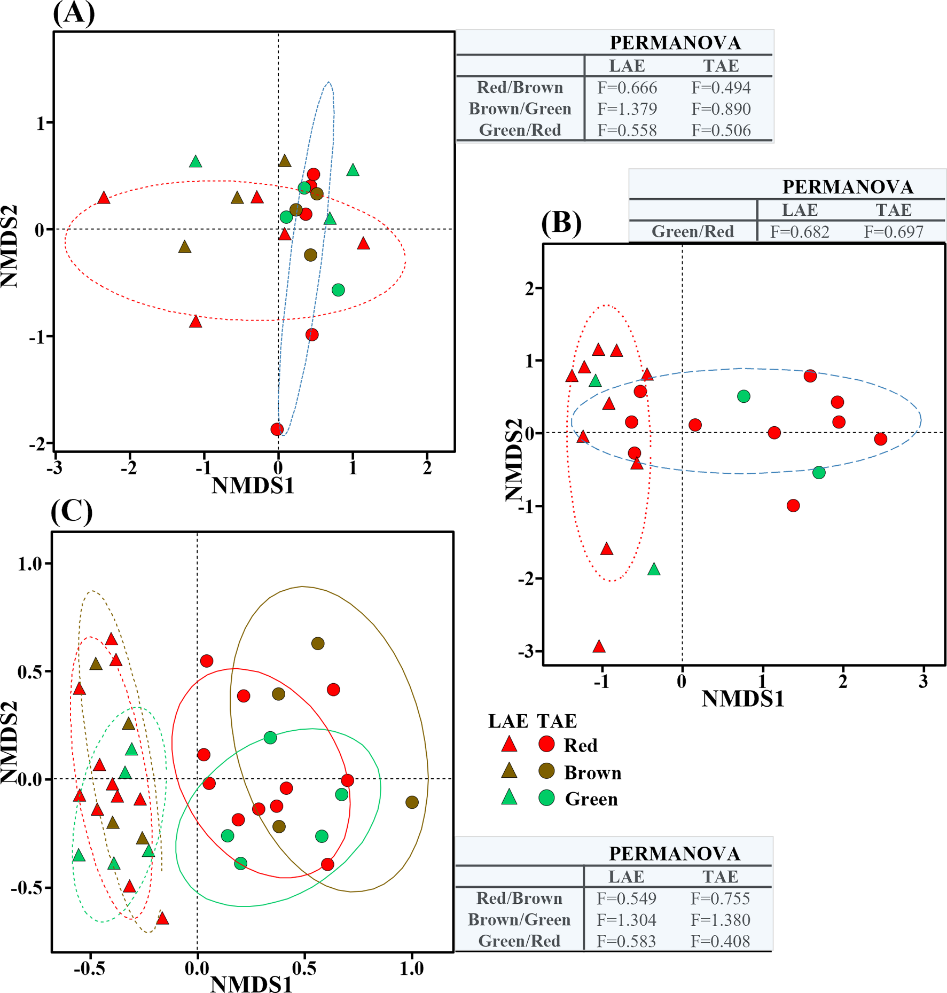


**Fig. S5** Non-metric multidimensional scaling (NMDS) plots comparing bacterial communities in the loosely attached environment (LAE) and tightly attached environment (TAE) of red (A), brown (B), and green (C) macroalgae from the East, West, and South Seas. Bacterial community dissimilarities based on location were assessed using the Bray-Curtis method, with statistical significance evaluated by PERMANOVA. Significant differences between groups are indicated by *p*-values < 0.05 (*) and < 0.001 (***).


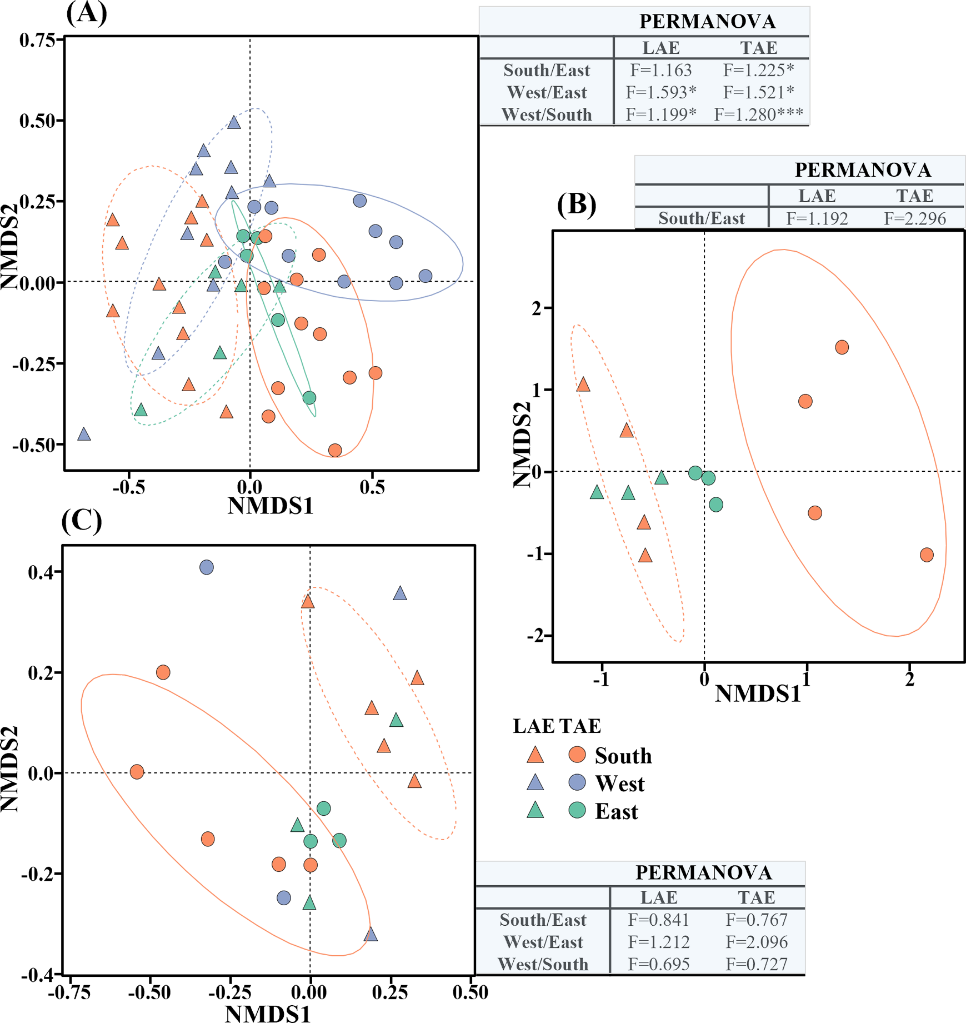


**Fig. S6** Phylogenetic trees showing the phylogenetic positions between strains RW-H-Ap-1 (CP176466–7, **A**), GE-S-Ul-11 (CP176468, **B**), RS-M-Aa-14 (CP176470, **C**), and RW-G-Af-16 (CP176469, **D**), and their closely related taxa, based on the concatenated sequences of 120 bacterial marker proteins. Bootstrap values (>70%) from 1000 replicate analyses are indicated at the branch points. Scale bars represent the number of changes per amino acid position. Pairwise average nucleotide identity (ANI) and digital DNA-DNA hybridization (dDDH) values were indicated using heatmaps on the right side.


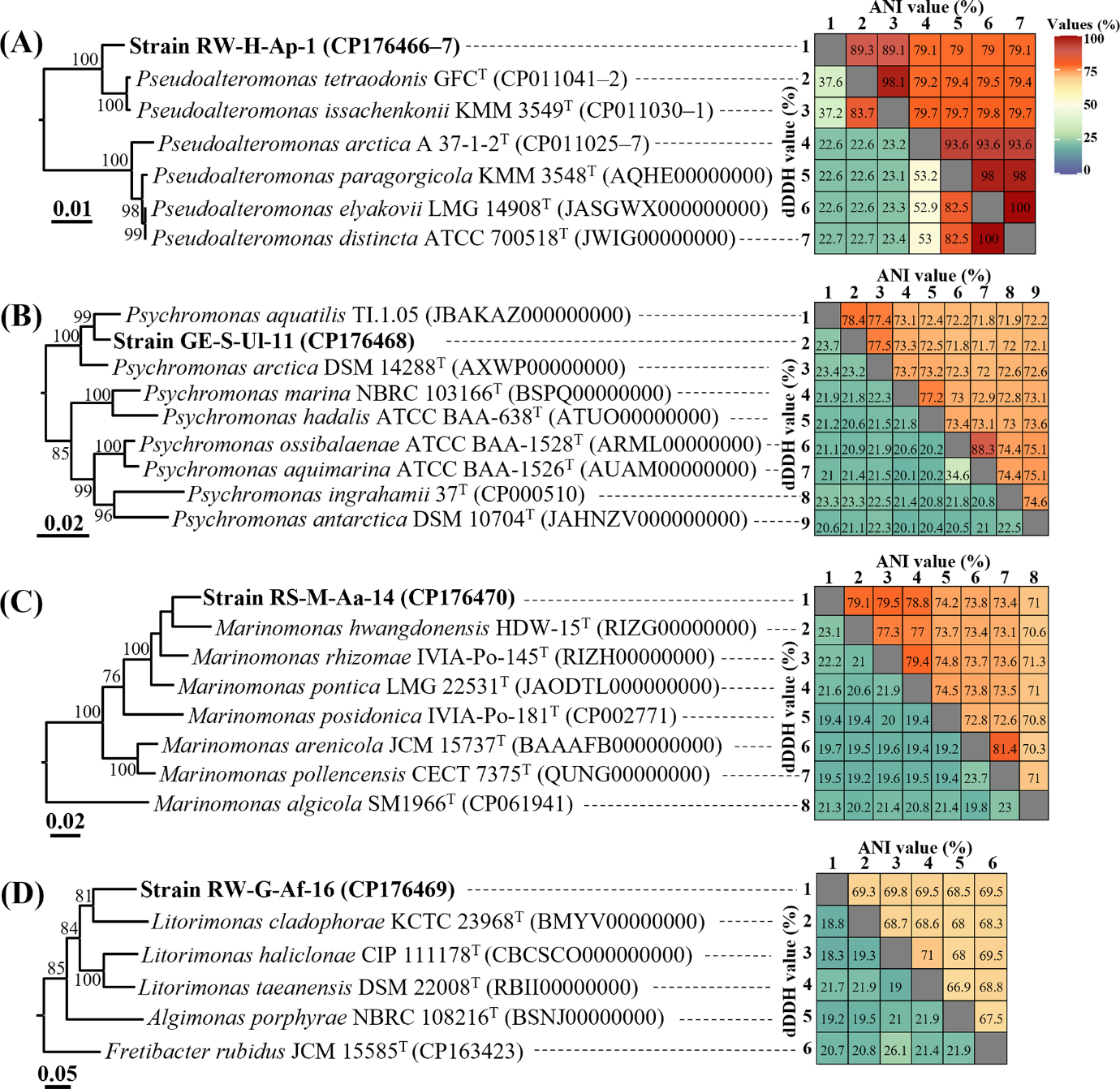


**Fig. S7** Metabolic pathways of phenylacetic acid (PAA) and 2-hydroxy-PAA (2-OH-PAA) from phenylalanine identified in potential keystone strains isolated from the phycospheres.

**
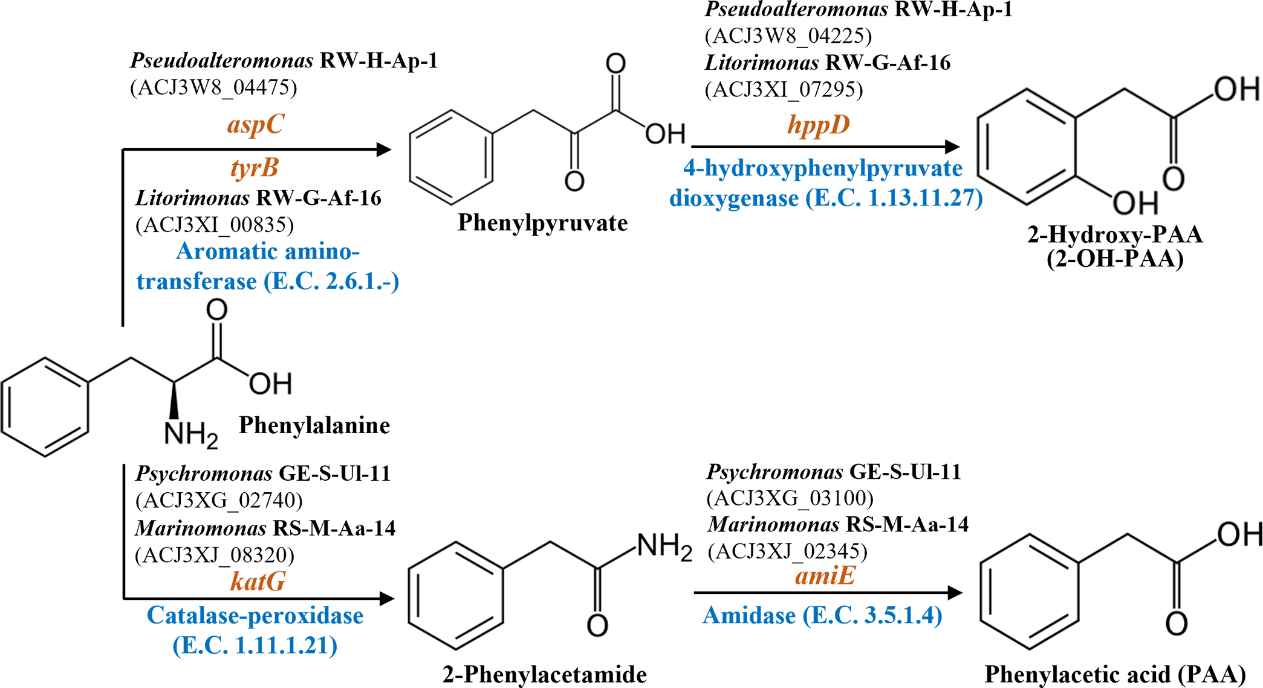
**

**Fig. S8** Demethylation pathway of dimethylsulfoniopropionate (DMSP) (**A**) and physical maps of the associated metabolic genes identified in the genomes of strains RS-M-Aa-14 and RW-G-Af-16 and their closely related type strains (**B**). The metabolic genes identified in strains RS-M-Aa-14 and RW-G-Af-16 are indicated within the demethylation pathway. MMPA, 3‐methylmercaptopropionate; MTA-CoA, methylthioacrylyl‐CoA.


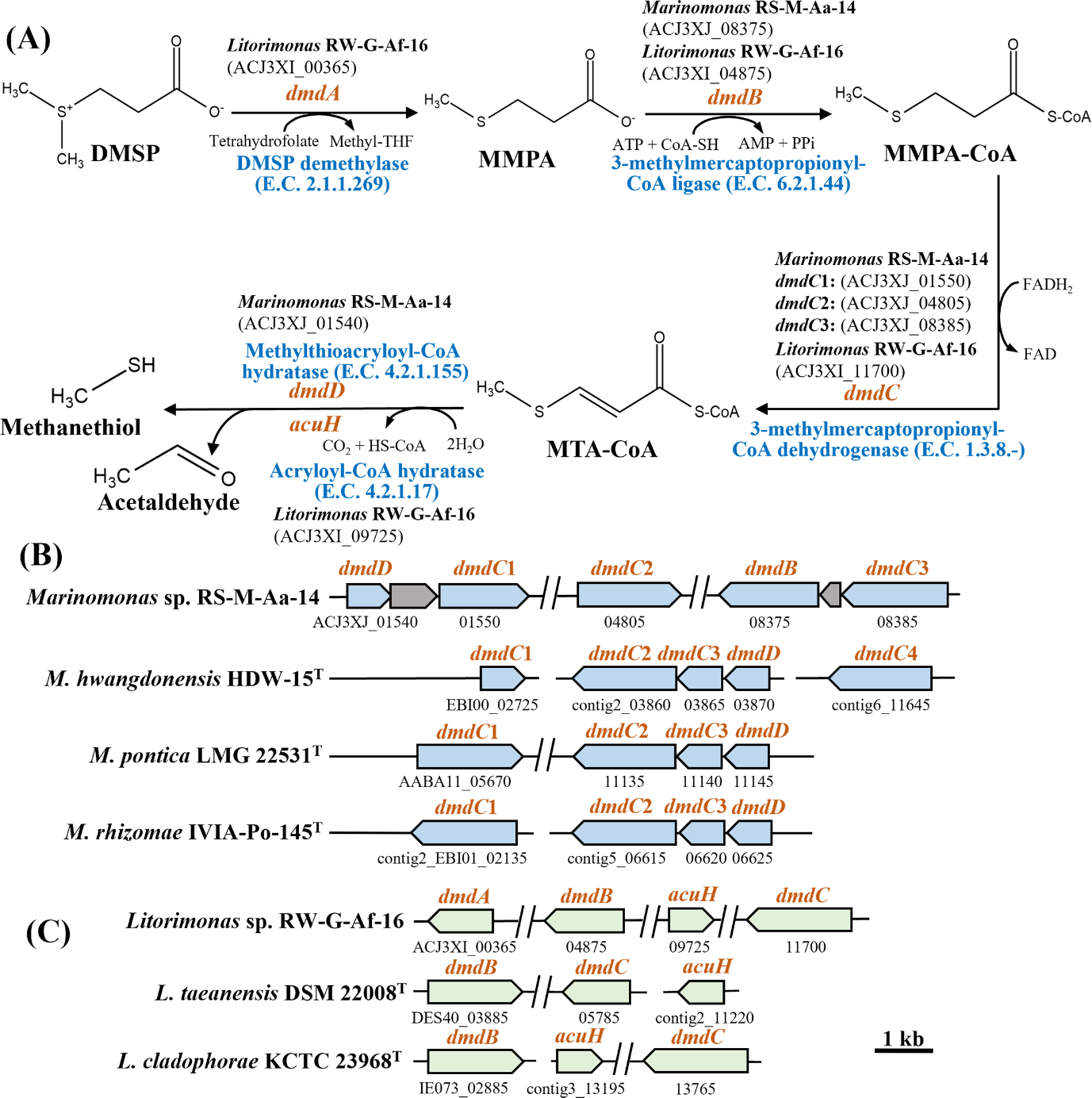


**Fig. S9** Biosynthesis pathway of zeaxanthin (**A**) and physical maps of the biosynthetic genes identified in the genomes of *Litorimonas* sp. RW-G-Af-16 and its closely related type strains (**B**). Biosynthetic genes identified in strain RW-G-Af-16 are indicated within the biosynthesis pathway.


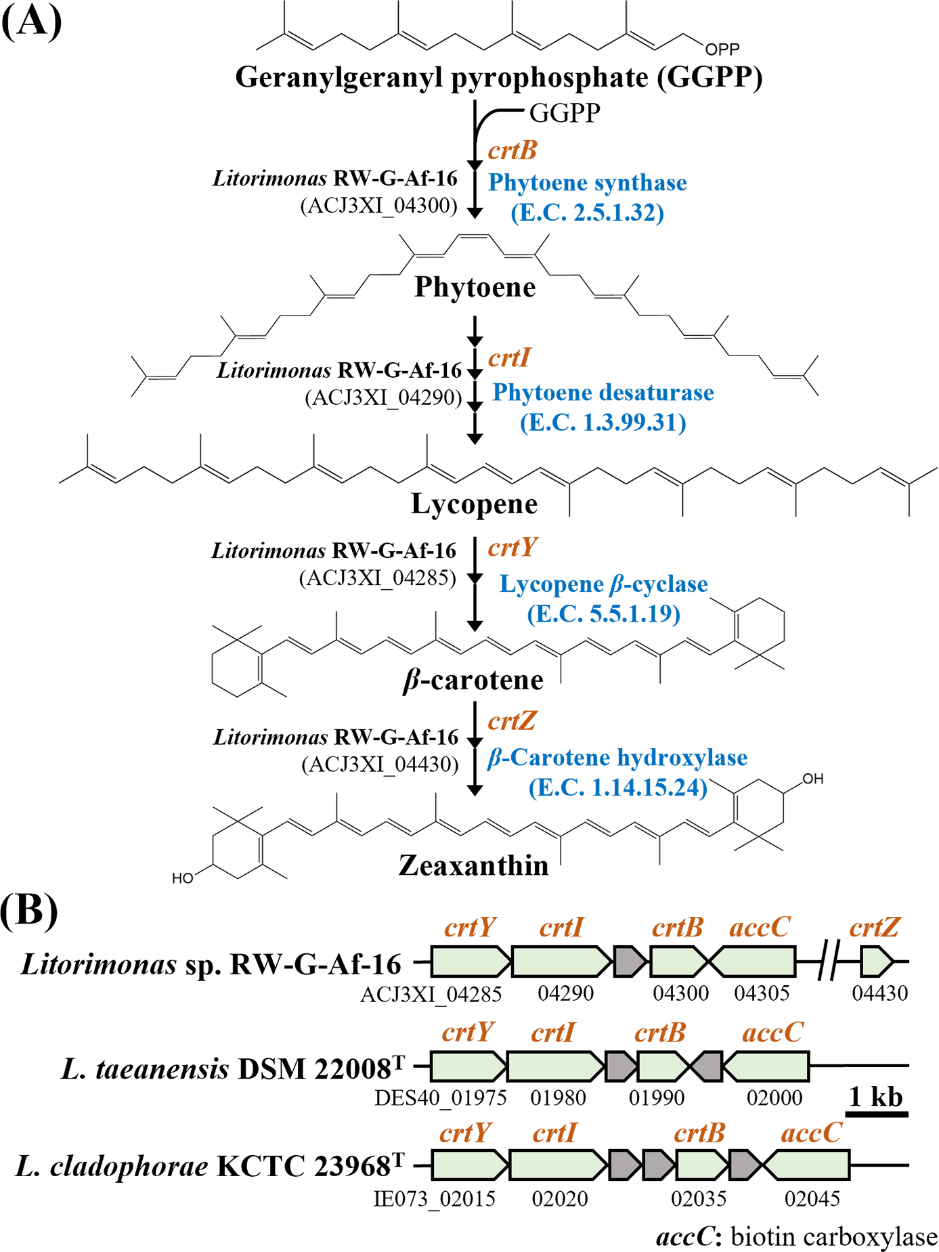


**Fig. S10** Physical map of a putative nitrogen-fixing gene cluster identified in *Litorimonas* sp. RW-G-Af-16.


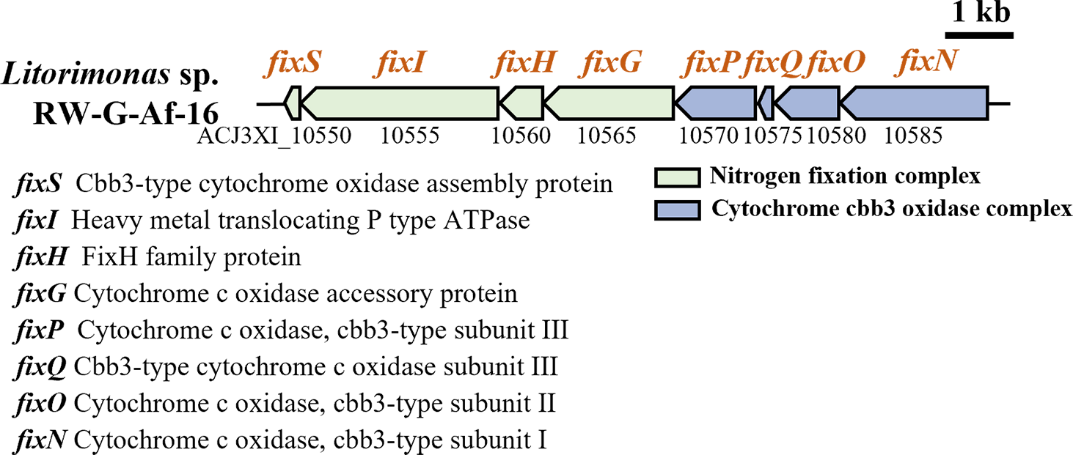


**Table S1** Taxonomic information and images of the marine macroalgae used for bacterial community analysis in this study. Scale bar, 2 cm.

| **Collection site** | **Color type** | **Taxon name*** | **Strain name**^†^ | **Image** |
| --- | --- | --- | --- | --- |
| South Sea (Mongdol) | Red | *Acrochaetium arcuatum* | RS-M-Aa | 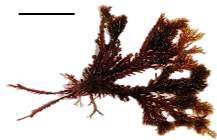 |
|  |  | *Dasysiphonia japonica* | RS-M-Dj | 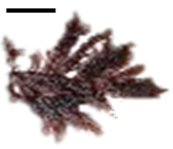 |
|  |  | *Neosiphonia yendoi* | RS-M-Ny | 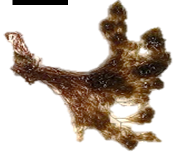 |
|  |  | *Ahnfeltiopsis flabelliformis* | RS-M-Af | 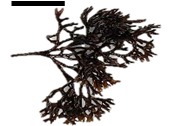 |
|  |  | *Grateloupia lanceolata* | RS-M-Gl | 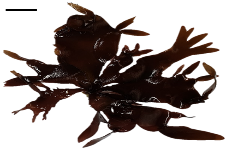 |
|  |  | *Besa catenata* | RS-M-Bc | 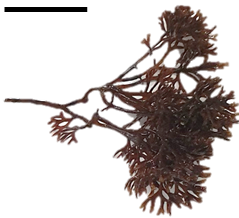 |
|  |  | *Pyropia suborbiculata* | RS-M-Ps | 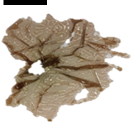 |
|  | Brown | *Sargassum miyabei* | BS-M-Sm | 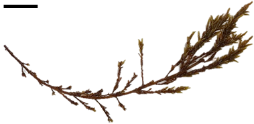 |
|  | Green | *Codium fragile* | GS-M-Cf | 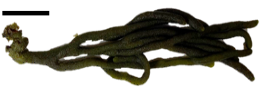 |
|  |  | *Chaetomorpha antennina* | GS-M-Ca | 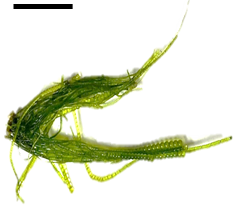 |
|  |  | *Ulva linza* | GS-M-Ul | 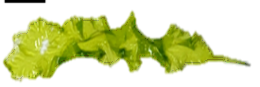 |
| South Sea (Nogu Port) | Red | *Grateloupia divaricata* | RS-N-Gd | 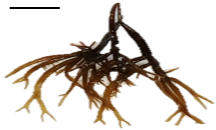 |
|  |  | *Besa catenata* | RS-N-Bc | 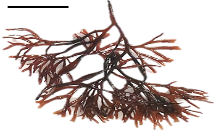 |
|  |  | *Schizymenia dubyi* | RS-N-Sd | 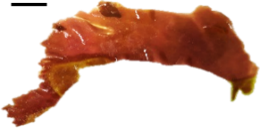 |
|  |  | *Symphyocladia linearis* | RS-N-Sl | 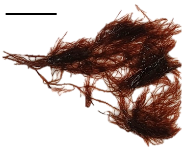 |
|  | Brown | *Sargassum thunbergii* | BS-N-St | 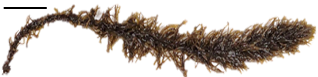 |
|  |  | *Saccharina japonica* | BS-N-Sj | 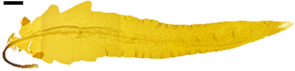 |
|  |  | *Sargassum fusiforme* | BS-N-Sf | 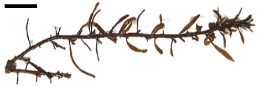 |
|  | Green | *Ulva australis* | GS-N-Ua | 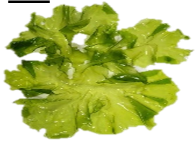 |
|  |  | *Chaetomorpha valida* | GS-N-Cv | 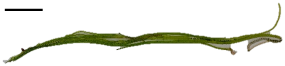 |
| West Sea (Guryepo) | Red | *Neorhodomela aculeata* | RW-G-Na | 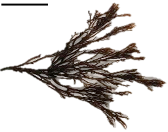 |
|  |  | *Ahnfeltiopsis flabelliformis* | RW-G-Af | 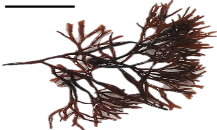 |
|  |  | *Bangia gloiopeltidicola* | RW-G-Bg | 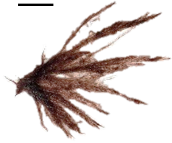 |
|  |  | *Gelidium elegans* | RW-G-Ge | 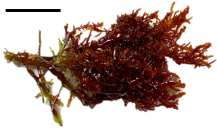 |
|  |  | *Besa catenata* | RW-G-Bc | 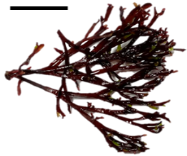 |
|  | Green | *Ulva australis* | GW-G-Ua | 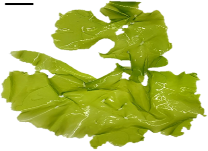 |
| West Sea (Hagampo) | Red | *Agarophyton vermiculophyllum* | RW-H-Av | 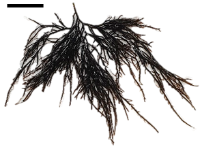 |
|  |  | *Callophyllis crispata* | RW-H-Cc | 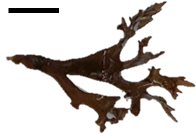 |
|  |  | *Lomentaria hakodatensis* | RW-H-Lh | 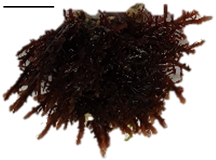 |
|  |  | *Grateloupia jejuensis* | RW-H-Gj | 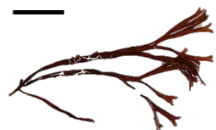 |
|  |  | *Acrochaetium plumosum* | RW-H-Ap | 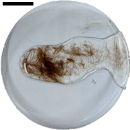 |
|  | Green | *Chaetomorpha linum* | GW-H-Cl | 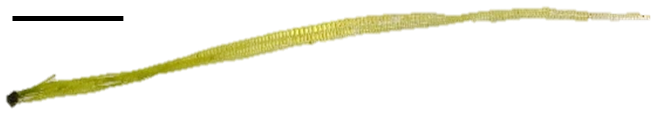 |
| East Sea (Galnam) | Red | *Chondracanthus intermedius* | RE-G-Ci | 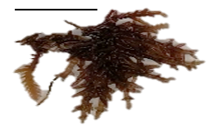 |
|  |  | *Besa divaricata* | RE-G-Bd | 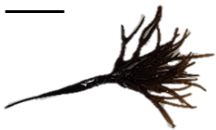 |
|  |  | *Pyropia suborbiculata* | RE-G-Ps | 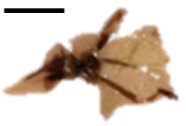 |
|  | Brown | *Sargassum vachellianum* | BE-G-Sv | 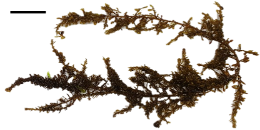 |
|  |  | *Feldmannia chitonicola* | BE-G-Fc | 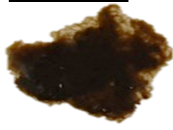 |
|  | Green | *Ulva australis* | GE-G-Ua | 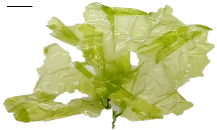 |
| East Sea (Sinnam) | Red | *Symphyocladia latiuscula* | RE-S-Sl | 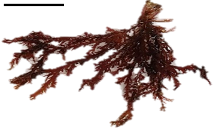 |
|  |  | *Grateloupia divaricata* | RE-S-Gd | 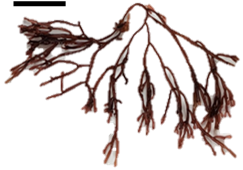 |
|  | Brown | *Ishige sinicola* | BE-S-Is | 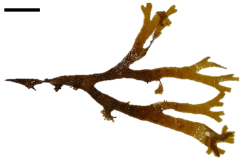 |
|  | Green | *Codium fragile* | GE-S-Cf | 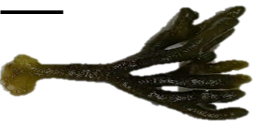 |
|  |  | *Ulva linza* | GE-S-Ul | 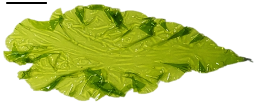 |

***** Marine macroalgae were taxonomically identified based on their *rbcL* sequences.

**Table S2** Summary and statistical diversity indices of bacterial 16S rRNA gene sequencing reads from loosely attached bacteria on marine algae, generated by paired-end Illumina MiSeq sequencing.

| **Marine algae** | | **Total reads** | **High quality reads^§^** | **OTU*** | **Shannon-Weaver** | **Chao1** | **Simpson** |
| --- | --- | --- | --- | --- | --- | --- | --- |
| South Sea | RS-M-Aa | 153,006 | 38,106 | 83 | 3.455 | 83.8 | 0.8 |
|  | RS-M-Dj | 123,711 | 35,623 | 223 | 5.399 | 223.0 | 0.9 |
|  | RS-M-Ny | 224,865 | 57,071 | 127 | 4.276 | 127.1 | 0.9 |
|  | RS-M-Af | 117,415 | 33,300 | 158 | 4.884 | 158.0 | 0.9 |
|  | RS-M-Gl | 107,092 | 22,920 | 135 | 5.262 | 136.0 | 0.9 |
|  | RS-M-Bc | 222,224 | 73,622 | 150 | 3.978 | 150.0 | 0.9 |
|  | RS-M-Ps | 109,741 | 29,500 | 188 | 5.415 | 188.2 | 0.9 |
|  | BS-M-Sm | 127,459 | 33,871 | 241 | 5.446 | 241.2 | 0.9 |
|  | GS-M-Cf | 166,919 | 50,830 | 248 | 5.019 | 248.0 | 0.9 |
|  | GS-M-Ca | 161,870 | 44,368 | 84 | 4.053 | 84.5 | 0.9 |
|  | GS-M-Ul | 167,473 | 45,574 | 181 | 4.995 | 181.6 | 0.9 |
|  | RS-N-Gd | 209,652 | 42,404 | 308 | 5.990 | 308.0 | 1.0 |
|  | RS-N-Bc | 172,680 | 35,216 | 130 | 3.920 | 131.0 | 0.8 |
|  | RS-N-Sd | 149,635 | 39,623 | 211 | 4.984 | 211.1 | 0.9 |
|  | RS-N-Sl | 121,997 | 27,433 | 93 | 4.681 | 93.0 | 0.9 |
|  | BS-N-St | 303,667 | 36,314 | 185 | 5.617 | 185.0 | 1.0 |
|  | BS-N-Sj | 126,418 | 36,580 | 188 | 4.913 | 188.0 | 0.9 |
|  | BS-N-Sf | 125,615 | 37,304 | 110 | 3.727 | 110.0 | 0.8 |
|  | GS-N-Ua | 79,913 | 13,554 | 63 | 2.830 | 63.0 | 0.6 |
|  | GS-N-Cv | 216,978 | 45,666 | 155 | 4.962 | 155.5 | 0.9 |
| West Sea | RW-G-Na | 179,432 | 65,451 | 390 | 4.696 | 390.0 | 0.9 |
|  | RW-G-Af | 132,515 | 56,707 | 20 | 1.177 | 20.0 | 0.4 |
|  | RW-G-Bg | 138,390 | 78,791 | 44 | 1.893 | 44.0 | 0.6 |
|  | RW-G-Ge | 204,068 | 51,064 | 133 | 4.828 | 133.0 | 0.9 |
|  | RW-G-Bc | 143,789 | 74,252 | 35 | 2.275 | 35.0 | 0.7 |
|  | GW-G-Ua | 172,361 | 53,088 | 183 | 5.015 | 183.9 | 0.9 |
|  | RW-H-Av | 83,296 | 32,154 | 120 | 3.420 | 120.0 | 0.8 |
|  | RW-H-Cc | 68,779 | 26,365 | 107 | 3.813 | 107.1 | 0.9 |
|  | RW-H-Lh | 89,387 | 20,628 | 95 | 4.912 | 95.0 | 0.9 |
|  | RW-H-Gj | 241,306 | 66,615 | 142 | 3.529 | 142.0 | 0.7 |
|  | RW-H-Ap | 261,964 | 111,311 | 240 | 3.271 | 240.0 | 0.7 |
|  | GW-H-Cl | 254,080 | 89,098 | 67 | 3.392 | 67.0 | 0.8 |
| East Sea | RE-G-Ci | 104,196 | 25,820 | 207 | 5.584 | 208.6 | 0.9 |
|  | RE-G-Bd | 116,434 | 31,818 | 164 | 5.504 | 164.0 | 1.0 |
|  | RE-G-Ps | 117,915 | 30,068 | 214 | 4.779 | 214.8 | 0.9 |
|  | BE-G-Sv | 239,712 | 78,794 | 399 | 5.172 | 399.8 | 0.9 |
|  | BE-G-Fc | 120,639 | 28,131 | 168 | 5.106 | 168.4 | 0.9 |
|  | GE-G-Ua | 118,929 | 26,353 | 195 | 5.864 | 195.1 | 1.0 |
|  | RE-S-Sl | 45,881 | 38,442 | 200 | 4.193 | 200.0 | 0.8 |
|  | RE-S-Gd | 131,629 | 42,088 | 228 | 5.181 | 228.2 | 0.9 |
|  | BE-S-Is | 123,667 | 40,238 | 212 | 5.385 | 212.3 | 0.9 |
|  | GE-S-Cf | 137,596 | 60,045 | 373 | 5.811 | 373.2 | 1.0 |
|  | GE-S-Ul | 102,874 | 30,382 | 119 | 3.578 | 119.0 | 0.8 |

**^§^**High-quality reads refer to sequencing reads that have been filtered to exclude low-quality and singleton amplicon sequence variants.

*OTUs (Operational Taxonomic Units) were defined based on a 99% sequence identity threshold.

**Table S3** Summary and statistical diversity indices of bacterial 16S rRNA gene sequencing reads from tightly attached bacteria on marine algae, generated by paired-end Illumina MiSeq sequencing.

| **Marine algae** | | **Total reads** | **High quality reads^§^** | **OTU*** | **Shannon-Weaver** | **Chao1** | **Simpson** |
| --- | --- | --- | --- | --- | --- | --- | --- |
| South Sea | RS-M-Aa | 77,882 | 66,353 | 201 | 3.218 | 201.0 | 0.7 |
|  | RS-M-Dj | 98,755 | 24,878 | 383 | 6.279 | 383.7 | 1.0 |
|  | RS-M-Ny | 121,754 | 40,280 | 225 | 4.269 | 225.0 | 0.9 |
|  | RS-M-Af | 118,764 | 56,272 | 134 | 3.909 | 134.0 | 0.8 |
|  | RS-M-Gl | 84,500 | 43,967 | 490 | 5.546 | 490.0 | 0.8 |
|  | RS-M-Bc | 463,447 | 75,261 | 794 | 7.470 | 794.3 | 1.0 |
|  | RS-M-Ps | 58,844 | 48,050 | 358 | 6.345 | 358.0 | 1.0 |
|  | BS-M-Sm | 53,793 | 43,585 | 369 | 6.402 | 369.6 | 1.0 |
|  | GS-M-Cf | 60,651 | 20,578 | 232 | 5.196 | 232.2 | 0.9 |
|  | GS-M-Ca | 82,237 | 30,793 | 280 | 5.545 | 280.0 | 0.9 |
|  | GS-M-Ul | 101,533 | 53,776 | 140 | 2.915 | 140.0 | 0.6 |
|  | RS-N-Gd | 72,036 | 13,930 | 234 | 6.394 | 234.1 | 1.0 |
|  | RS-N-Bc | 69,098 | 23,302 | 212 | 4.312 | 212.1 | 0.8 |
|  | RS-N-Sd | 58,962 | 25,302 | 394 | 6.725 | 394.0 | 1.0 |
|  | RS-N-Sl | 88,267 | 75,013 | 244 | 2.961 | 244.2 | 0.5 |
|  | BS-N-St | 127,926 | 53,912 | 186 | 1.750 | 186.4 | 0.3 |
|  | BS-N-Sj | 83,017 | 36,177 | 421 | 6.696 | 421.3 | 1.0 |
|  | BS-N-Sf | 82,836 | 59,216 | 243 | 3.216 | 243.0 | 0.6 |
|  | GS-N-Ua | 65,483 | 33,588 | 485 | 7.900 | 485.1 | 1.0 |
|  | GS-N-Cv | 148,575 | 24,966 | 195 | 5.931 | 195.0 | 1.0 |
| West Sea | RW-G-Na | 47,674 | 32,628 | 241 | 6.505 | 241.0 | 1.0 |
|  | RW-G-Af | 69,808 | 37,308 | 237 | 5.184 | 237.0 | 0.9 |
|  | RW-G-Bg | 239,470 | 38,666 | 258 | 5.446 | 258.0 | 1.0 |
|  | RW-G-Ge | 75,777 | 64,996 | 173 | 4.772 | 173.0 | 0.9 |
|  | RW-G-Bc | 329,260 | 13,729 | 61 | 4.364 | 61.0 | 0.9 |
|  | GW-G-Ua | 66,740 | 48,111 | 235 | 4.811 | 235.0 | 0.9 |
|  | RW-H-Av | 78,104 | 31,569 | 185 | 4.249 | 185.0 | 0.8 |
|  | RW-H-Cc | 203,410 | 30,303 | 161 | 5.321 | 161.0 | 1.0 |
|  | RW-H-Lh | 110,274 | 22,076 | 240 | 5.915 | 240.0 | 1.0 |
|  | RW-H-Gj | 338,691 | 25,513 | 192 | 5.242 | 192.0 | 0.9 |
|  | RW-H-Ap | 70,444 | 30,433 | 80 | 3.331 | 80.3 | 0.7 |
|  | GW-H-Cl | 209,691 | 44,015 | 150 | 4.406 | 150.0 | 0.9 |
| East Sea | RE-G-Ci | 120,983 | 33,407 | 195 | 4.948 | 195.0 | 0.9 |
|  | RE-G-Bd | 40,295 | 34,659 | 108 | 3.769 | 108.0 | 0.8 |
|  | RE-G-Ps | 772,622 | 37,142 | 287 | 5.477 | 287.4 | 0.9 |
|  | BE-G-Sv | 689,430 | 12,285 | 89 | 4.718 | 89.0 | 0.9 |
|  | BE-G-Fc | 88,523 | 26,409 | 166 | 4.081 | 166.0 | 0.8 |
|  | GE-G-Ua | 94,165 | 38,995 | 148 | 3.512 | 148.1 | 0.8 |
|  | RE-S-Sl | 62,828 | 44,122 | 325 | 5.771 | 325.4 | 0.9 |
|  | RE-S-Gd | 80,545 | 39,749 | 159 | 3.047 | 159.0 | 0.6 |
|  | BE-S-Is | 41,207 | 34,819 | 173 | 4.657 | 173.0 | 0.9 |
|  | GE-S-Cf | 72,320 | 37,879 | 331 | 6.073 | 331.2 | 0.9 |
|  | GE-S-Ul | 61,730 | 47,086 | 251 | 5.677 | 251.0 | 0.9 |

**^§^**High-quality reads refer to sequencing reads that have been filtered to exclude low-quality and singleton amplicon sequence variants.

*OTUs (Operational Taxonomic Units) were defined based on a 99% sequence identity threshold.

**Table S4** Summary and statistical diversity indices of bacterial 16S rRNA gene sequencing reads from seawater where the marine algae were collected, generated by paired-end Illumina MiSeq sequencing.

| **Seawater** | | **Total reads** | **High quality reads^§^** | **OTU*** | **Shannon-Weaver** | **Chao1** | **Simpson** |
| --- | --- | --- | --- | --- | --- | --- | --- |
| South Sea | Mongdol | 55,703 | 17,408 | 207 | 5.321 | 207.3 | 0.9 |
|  | Nogu Port | 47,133 | 14,569 | 189 | 6.000 | 189.0 | 1.0 |
| West Sea | Guryepo | 206,447 | 31,084 | 891 | 6.570 | 893.4 | 1.0 |
|  | Hagampo | 65,669 | 31,009 | 444 | 7.065 | 444.0 | 1.0 |
| East Sea | Galnam | 63,691 | 21,535 | 206 | 5.046 | 206.5 | 0.9 |
|  | Sinnam | 58,047 | 20,560 | 195 | 5.338 | 195.1 | 0.9 |

**^§^**High-quality reads refer to sequencing reads that have been filtered to exclude low-quality and singleton amplicon sequence variants.

*OTUs (Operational Taxonomic Units) were defined based on a 99% sequence identity threshold.

**Table S5** Lists of bacterial strains isolated from the phycosphere of marine macroalgae, each exhibiting distinct 16S rRNA gene fragment patterns. These strains were taxonomically classified and assigned to the closest species based on 16S rRNA gene sequence similarities using the Nucleotide Similarity Search program (http://www.ezbiocloud.net/identify/). Among them, four strains (highlighted in bold) were selected for whole-genome sequencing to investigate their metabolic features involved in interactions with marine macroalgae.

| **Strain** | **Top-hit taxon type strain** | **Similarity (%)** |
| --- | --- | --- |
| **Phylum *Pseudomonadota* (211 strains)** | | |
| RS-N-Bc-2 | *Actibacterium pelagium* JN33^T^ | 97.94 |
| GS-M-Ul-11 | *Actibacterium pelagium* JN33^T^ | 96.88 |
| GE-S-Cf-9 | *Aestuariibius insulae* DBTF-13^T^ | 96.77 |
| RS-N-Sd-15 | *Aliishimia ponticola* MYP11^T^ | 97.18 |
| RE-S-Gd-16 | *Aliishimia ponticola* MYP11^T^ | 96.37 |
| BS-N-St-6 | *Altererythrobacter insulae* BPTF-M16^T^ | 97.32 |
| RW-G-Na-9 | *Altererythrobacter ishigakiensis* ATCC BAA-2084^T^ | 97.73 |
| RS-N-Gf-12 | *Altererythrobacter ishigakiensis* ATCC BAA-2084^T^ | 97.73 |
| RS-M-Ny-17 | *Altererythrobacter rubellus* KMU-45^T^ | 99.50 |
| RW-G-Bg-16 | *Alteromonas addita* R10SW13^T^ | 100 |
| RS-M-Aa-6 | *Alteromonas addita* R10SW13^T^ | 100 |
| GE-S-Ul-8 | *Alteromonas addita* R10SW13^T^ | 100 |
| RW-G-Bg-21 | *Alteromonas stellipolaris* LMG 21861^T^ | 99.79 |
| RS-M-Ps-1 | *Aurantiacibacter aquimixticola* JSSK-14^T^ | 98.78 |
| RS-N-Av-3 | *Aurantiacibacter gangjinensis* K7-2^T^ | 99.72 |
| RS-N-Sl-13 | *Cognatiyoonia koreensis* DSM 17925^T^ | 98.29 |
| RS-N-Av-2 | *Cognatiyoonia koreensis* DSM 17925^T^ | 98.05 |
| GS-M-Ul-1 | *Cognatiyoonia koreensis* DSM 17925^T^ | 97.69 |
| BS-M-Sm-3 | *Cognatiyoonia koreensis* DSM 17925^T^ | 97.33 |
| RS-N-Gf-10 | *Cognatiyoonia sediminum* DSM 28715^T^ | 97.54 |
| GS-M-Ca-1 | *Cognatiyoonia sediminum* DSM 28715^T^ | 98.48 |
| RE-S-Sl-9 | *Colwellia agarivorans* QM50^T^ | 99.45 |
| RE-G-Ci-8 | *Denitrobaculum tricleocarpae* R148^T^ | 100 |
| RS-M-Ps-4 | *Erythrobacter insulae* JBTF-M21^T^ | 98.86 |
| RS-M-Gf-1 | *Erythrobacter longus* DSM 6997^T^ | 99.82 |
| RS-M-Dj-1 | *Erythrobacter longus* DSM 6997^T^ | 98.65 |
| RS-M-Bc-11 | *Erythrobacter longus* DSM 6997^T^ | 99.89 |
| RS-M-Af-4 | *Erythrobacter longus* DSM 6997^T^ | 98.96 |
| GE-S-Cf-8 | *Erythrobacter longus* DSM 6997^T^ | 98.77 |
| RW-G-Bg-12 | *Glaciecola nitratireducens* FR1064^T^ | 96.15 |
| RW-H-Gj-16 | *Glaciecola punicea* ACAM 611^T^ | 96.23 |
| RS-M-Bc-7 | *Glaciecola punicea* ACAM 611^T^ | 95.73 |
| RE-S-Sl-5 | *Glaciecola punicea* ACAM 611^T^ | 96.01 |
| BS-N-Is-8 | *Hellea balneolensis* DSM 19091^T^ | 96.57 |
| BS-N-Is-9 | *Hyphomonas oceanitis* SCH89^T^ | 94.68 |
| RS-N-Sd-8 | *Jannaschia aquimarina* GSW-M26^T^ | 97.55 |
| BS-N-Sj-3 | *Jannaschia donghaensis* CECT 7802^T^ | 99.16 |
| RS-M-Aa-1 | *Jannaschia faecimaris* DSM 100420^T^ | 99.50 |
| RS-N-Gf-17 | *Jannaschia helgolandensis* DSM 14858^T^ | 96.97 |
| BS-N-Sj-7 | *Jannaschia helgolandensis* DSM 14858^T^ | 97.49 |
| RS-N-Av-5 | *Jannaschia rubra* CECT 5088^T^ | 97.04 |
| RS-M-Dj-9 | *Jannaschia rubra* CECT 5088^T^ | 97.76 |
| BS-N-Is-4 | *Jannaschia seohaensis* DSM 25227^T^ | 99.06 |
| RS-N-Gf-4 | *Lentilitoribacter donghaensis* BH-4^T^ | 98.41 |
| RE-G-Ps-8 | *Lentilitoribacter donghaensis* BH-4^T^ | 98.97 |
| BS-N-Sj-4 | *Lentilitoribacter donghaensis* BH-4^T^ | 99.16 |
| RW-G-Ge-8 | *Litoreibacter albidus* DSM 26922^T^ | 99.81 |
| RS-M-Af-6 | *Litoreibacter albidus* DSM 26922^T^ | 99.27 |
| RE-G-Pc-3 | *Litoreibacter albidus* DSM 26922^T^ | 99.16 |
| GS-M-Ul-13 | *Litoreibacter albidus* DSM 26922^T^ | 98.41 |
| GS-M-Cf-2 | *Litoreibacter albidus* DSM 26922^T^ | 98.56 |
| GE-G-Ua-4 | *Litoreibacter albidus* DSM 26922^T^ | 98.97 |
| BS-N-St-2 | *Litoreibacter albidus* DSM 26922^T^ | 99.23 |
| RS-N-Sd-6 | *Litoreibacter halocynthiae* DSM 29467^T^ | 97.47 |
| RS-N-Gf-18 | *Litoreibacter halocynthiae* DSM 29467^T^ | 98.48 |
| GS-M-Cf-3 | *Litoreibacter halocynthiae* DSM 29467^T^ | 98.97 |
| RS-N-Gf-8 | *Litoreibacter janthinus* DSM 26921^T^ | 99.34 |
| RE-S-Sl-14 | *Litoreibacter janthinus* DSM 26921^T^ | 99.22 |
| RE-G-Ci-5 | *Litoreibacter janthinus* DSM 26921^T^ | 98.85 |
| BE-G-Fc-2 | *Litoreibacter janthinus* DSM 26921^T^ | 99.38 |
| RS-N-Sl-2 | *Litoreibacter meonggei* DSM 29466^T^ | 95.96 |
| BS-N-Sj-15 | *Litoreibacter meonggei* DSM 29466^T^ | 99.27 |
| RW-H-Gj-8 | *Litoreibacter ponti* GJSW-31^T^ | 98.92 |
| RS-N-Sd-7 | *Litoreibacter ponti* GJSW-31^T^ | 99.44 |
| RS-M-Dj-2 | *Litoreibacter ponti* GJSW-31^T^ | 98.27 |
| **RW-G-Af-16** | ***Litorimonas taeanensis* KACC 13701^T^** | **96.79** |
| RW-H-Av-2 | *Loktanella acticola* OISW-6^T^ | 98.18 |
| RS-N-Gd-18 | *Loktanella acticola* OISW-6^T^ | 98.32 |
| RS-M-Dj-4 | *Loktanella acticola* OISW-6^T^ | 99.63 |
| RS-M-Bc-2 | *Loktanella acticola* OISW-6^T^ | 98.29 |
| GW-G-Ua-2 | *Loktanella acticola* OISW-6^T^ | 98.31 |
| GE-G-Ua-3 | *Loktanella acticola* OISW-6^T^ | 98.74 |
| BE-G-Sv-1 | *Loktanella acticola* OISW-6^T^ | 99.79 |
| BE-G-Fc-8 | *Loktanella acticola* OISW-6^T^ | 98.97 |
| RS-M-Af-22 | *Loktanella agnita* R10SW5^T^ | 98.08 |
| RS-N-Gf-11 | *Loktanella ponticola* SW2^T^ | 96.74 |
| BS-N-Sm-2 | *Loktanella ponticola* SW2^T^ | 97.18 |
| BS-N-Is-12 | *Loktanella ponticola* SW2^T^ | 96.74 |
| RW-H-Gj-14 | *Marinicella sediminis* F2^T^ | 94.00 |
| **RS-M-Aa-14** | ***Marinomonas algicola* SM1966^T^** | **99.66** |
| RS-M-Aa-20 | *Marinomonas arenicola* KMM 3893^T^ | 100 |
| GE-S-Ul-3 | *Marinomonas dokdonensis* DSW10-10^T^ | 99.62 |
| RS-M-Bc-5 | *Marinomonas gallaica* Cmf 17.2^T^ | 99.73 |
| RS-M-Ps-10 | *Marivita cryptomonadis* CL-SK44^T^ | 97.42 |
| RW-H-Lh-11 | *Marivita geojedonensis* DPG-138^T^ | 98.40 |
| BS-N-Is-11 | *Mesorhizobium sediminum* YIM M12096^T^ | 85.56 |
| RS-N-Av-1 | *Octadecabacter ascidiaceicola* CECT 8868^T^ | 98.86 |
| GS-M-Ul-2 | *Octadecabacter ascidiaceicola* CECT 8868^T^ | 97.84 |
| GE-G-Ua-9 | *Octadecabacter ascidiaceicola* CECT 8868^T^ | 98.82 |
| BS-N-Sm-8 | *Octadecabacter ascidiaceicola* CECT 8868^T^ | 98.27 |
| BE-S-Is-1 | *Octadecabacter ascidiaceicola* CECT 8868^T^ | 98.85 |
| GE-S-Ul-6 | *Paraglaciecola chathamensis* S18K6^T^ | 100 |
| RS-N-Sd-11 | *Parasphingorhabdus flavimaris* SW-151^T^ | 98.37 |
| RS-M-Ny-47 | *Parasphingorhabdus flavimaris* SW-151^T^ | 95.53 |
| RS-M-Ny-21 | *Parasphingorhabdus litoris* FR1093^T^ | 99.10 |
| RE-G-Ci-3 | *Parasphingorhabdus litoris* FR1093^T^ | 100 |
| BS-N-St-3 | *Parasphingorhabdus litoris* FR1093^T^ | 99.90 |
| RS-M-Ny-10 | *Parasphingorhabdus marina* DSM 22363^T^ | 99.27 |
| RS-N-Gf-2 | *Paraurantiacibacter namhicola* JCM 16345^T^ | 95.61 |
| RW-G-Ge-18 | *Parerythrobacter jejuensis* CNU001^T^ | 99.38 |
| GE-G-Ua-10 | *Pelagihabitans pacificus* TP-CH-4^T^ | 96.88 |
| RE-G-Ci-1 | *Phaeobacter inhibens* DSM 16374^T^ | 100 |
| RS-M-Af-19 | *Phaeobacter porticola* P97^T^ | 97.33 |
| RE-S-Sl-12 | *Planktotalea arctica* IMCC9565^T^ | 99.40 |
| RS-N-Gf-9 | *Pontixanthobacter aestiaquae* HDW-31^T^ | 97.16 |
| RS-M-Ny-34 | *Pontixanthobacter aquaemixtae* JSSK-8^T^ | 97.81 |
| RS-N-Gf-14 | *Pontixanthobacter luteolus* SW-109^T^ | 97.38 |
| RS-N-Gf-5 | *Primorskyibacter aestuariivivens* OITF-36^T^ | 97.40 |
| GE-S-Cf-7 | *Pseudahrensia todarodis* KHS02^T^ | 98.85 |
| BS-N-Sm-3 | *Pseudahrensia todarodis* KHS02^T^ | 99.60 |
| **RW-H-Ap-1** | ***Pseudoalteromonas atlantica* NBRC 103033^T^** | **99.93** |
| GE-S-Ul-4 | *Pseudoalteromonas atlantica* NBRC 103033^T^ | 100 |
| GS-N-Ua-1 | *Pseudooctadecabacter jejudonensis* SSK2-1^T^ | 98.48 |
| BS-N-Is-5 | *Pseudooctadecabacter jejudonensis* SSK2-1^T^ | 98.63 |
| RE-S-Sl-13 | *Psychromonas arctica* DSM 14288^T^ | 99.63 |
| GE-S-Cf-16 | *Psychromonas arctica* DSM 14288^T^ | 99.63 |
| **GE-S-Ul-11** | ***Psychromonas arctica* DSM 14288^T^** | **99.39** |
| RS-M-Gl-10 | *Psychrosphaera aestuarii* PSC101^T^ | 100 |
| RW-H-Lh-13 | *Qipengyuania algicida* Yeonmyeong 2-22^T^ | 98.20 |
| RW-H-Gf-11 | *Qipengyuania algicida* Yeonmyeong 2-22^T^ | 97.69 |
| GW-G-Ua-9 | *Qipengyuania algicida* Yeonmyeong 2-22^T^ | 97.70 |
| RW-G-Af-6 | *Qipengyuania aquimaris* SW-110^T^ | 99.28 |
| RS-N-Sd-4 | *Qipengyuania aquimaris* SW-110^T^ | 98.79 |
| RS-M-Mh-4 | *Qipengyuania aquimaris* SW-110^T^ | 98.87 |
| RS-M-Dj-10 | *Qipengyuania aquimaris* SW-110^T^ | 98.37 |
| RS-M-Ny-5 | *Qipengyuania citrea* RE35F/1^T^ | 98.51 |
| BS-N-Is-10 | *Qipengyuania citrea* RE35F/1^T^ | 98.99 |
| RS-N-Gd-7 | *Qipengyuania gaetbuli* SW-161^T^ | 98.14 |
| RW-G-Af-2 | *Qipengyuania nanhaisediminis* CGMCC 1.7715^T^ | 98.87 |
| RS-N-Gf-6 | *Qipengyuania nanhaisediminis* CGMCC 1.7715^T^ | 98.72 |
| RW-G-Af-13 | *Qipengyuania seohaensis* SW-135^T^ | 97.65 |
| RS-N-Gd-19 | *Qipengyuania seohaensis* SW-135^T^ | 97.73 |
| GE-S-Cf-5 | *Qipengyuania seohaensis* SW-135^T^ | 97.67 |
| RS-M-Ps-5 | Qi*pengyuania vulgaris* 022 2-10^T^ | 98.86 |
| RS-N-Gd-8 | *Rhodophyticola porphyridii* MA-7-27^T^ | 97.78 |
| BS-M-Sm-1 | *Robertmurraya kyonggiensis* NB22^T^ | 99.10 |
| RE-S-Gd-5 | *Roseibium aggregatum* IAM 12614^T^ | 99.69 |
| RS-N-Gd-14 | *Roseibium album* CECT 5094^T^ | 99.35 |
| RS-M-Mh-2 | *Roseibium album* CECT 5094^T^ | 98.86 |
| RE-G-Ps-1 | *Roseibium album* CECT 5094^T^ | 98.50 |
| RS-M-Ps-8 | *Roseibium alexandrii* DFL-11^T^ | 99.07 |
| BS-N-Sm-11 | *Roseibium alexandrii* DFL-11^T^ | 100 |
| BS-N-St-4 | *Roseibium hamelinense* ATCC BAA-252^T^ | 98.66 |
| RW-H-Gf-4 | *Roseobacter cerasinus* AI77^T^ | 100 |
| RS-N-Sd-10 | *Roseobacter cerasinus* AI77^T^ | 97.70 |
| RS-M-Dj-8 | *Roseobacter cerasinus* AI77^T^ | 99.13 |
| GE-G-Ua-7 | *Roseobacter cerasinus* AI77^T^ | 99.48 |
| BS-N-Sm-17 | *Roseobacter cerasinus* AI77^T^ | 99.47 |
| BS-N-Sj-12 | *Roseobacter cerasinus* AI77^T^ | 99.49 |
| BS-N-Sf-10 | *Roseobacter cerasinus* AI77^T^ | 98.24 |
| BS-N-St-11 | *Roseobacter denitrificans* OCh 114^T^ | 98.18 |
| RS-N-Sd-1 | *Roseobacter ponti* MM-7^T^ | 97.90 |
| RS-N-Gd-20 | *Roseovarius aestuarii* CECT 7745^T^ | 98.84 |
| RE-G-Pc-4 | *Roseovarius aestuarii* CECT 7745^T^ | 98.96 |
| GE-S-Cf-2 | *Roseovarius aestuarii* CECT 7745^T^ | 99.28 |
| GE-S-Cf-12 | *Roseovarius aestuariivivens* KX641473^T^ | 98.05 |
| RS-N-Sd-2 | *Roseovarius nubinhibens* ISM^T^ | 97.12 |
| RS-M-Ny-19 | *Roseovarius nubinhibens* ISM^T^ | 97.05 |
| RS-N-Gd-5 | *Ruegeria atlantica* CECT 4292^T^ | 99.83 |
| GE-S-Cf-3 | *Ruegeria atlantica* CECT 4292^T^ | 100 |
| BS-N-Sj-11 | *Ruegeria atlantica* CECT 4292^T^ | 99.36 |
| BS-N-Sv-1 | *Ruegeria conchae* TW15^T^ | 99.91 |
| RS-N-Sd-14 | *Ruegeria litorea* CECT 7639^T^ | 97.11 |
| BS-N-St-1 | *Ruegeria litorea* CECT 7639^T^ | 99.64 |
| RS-M-Mh-3 | *Ruegeria profundi* ZGT108^T^ | 98.97 |
| RS-M-Ps-11 | *Salaquimonas pukyongi* RR3-28^T^ | 95.38 |
| RS-N-Bc-3 | *Sedimentitalea todarodis* KHS03^T^ | 97.12 |
| GE-S-Ul-1 | *Shewanella inventionis* KX27^T^ | 98.76 |
| BS-N-Is-6 | *Shewanella japonica* KCTC 22435^T^ | 99.64 |
| RS-M-Ny-9 | *Shimia aestuarii* DSM 15283^T^ | 99.80 |
| RS-M-Mh-5 | *Shimia aestuarii* DSM 15283^T^ | 96.24 |
| BS-N-Is-3 | *Shimia sediminis* ZQ172^T^ | 97.19 |
| RS-M-Mh-1 | *Sphingorhabdus arenilitoris* GJR-7^T^ | 95.26 |
| RE-S-Sl-11 | *Sulfitobacter donghicola* KCTC 12864^T^ | 100 |
| GW-H-Cl-7 | *Sulfitobacter donghicola* KCTC 12864^T^ | 100 |
| RS-N-Gf-19 | *Sulfitobacter faviae* S5-53^T^ | 97.04 |
| RS-N-Gd-12 | *Sulfitobacter geojensis* MM-124^T^ | 99.15 |
| RS-M-Aa-4 | *Sulfitobacter geojensis* MM-124^T^ | 100 |
| GS-M-Ul-12 | *Sulfitobacter mediterraneus* KCTC 32188^T^ | 98.19 |
| GS-M-Ul-8 | *Sulfitobacter noctilucae* NB-68^T^ | 97.25 |
| RW-H-Gj-12 | *Sulfitobacter noctilucicola* NB-77^T^ | 99.32 |
| GE-G-Ua-14 | *Sulfitobacter noctilucicola* NB-77^T^ | 98.19 |
| BS-N-St-8 | *Sulfitobacter noctilucicola* NB-77^T^ | 99.37 |
| RE-G-Ci-4 | *Sulfitobacter pontiacus* DSM 10014^T^ | 98.01 |
| RS-N-Sd-3 | *Tateyamaria armeniaca* KMU-156^T^ | 97.25 |
| RS-N-Gd-6 | *Tateyamaria armeniaca* KMU-156^T^ | 99.15 |
| RS-M-Ps-6 | *Tateyamaria armeniaca* KMU-156^T^ | 98.96 |
| RS-M-Ny-35 | *Tateyamaria armeniaca* KMU-156^T^ | 97.53 |
| GE-S-Cf-1 | *Tateyamaria armeniaca* KMU-156^T^ | 97.92 |
| RS-M-Ps-9 | *Tateyamaria omphalii* MKT107^T^ | 99.42 |
| RE-G-Ci-9 | *Tateyamaria omphalii* MKT107^T^ | 99.38 |
| GE-S-Cf-10 | *Tateyamaria omphalii* MKT107^T^ | 98.24 |
| GS-M-Cf-1 | *Tateyamaria pelophila* SAM4^T^ | 91.79 |
| BS-N-Is-7 | *Tateyamaria pelophila* SAM4^T^ | 90.73 |
| RW-G-Ge-1 | *Thalassobius litorarius* MME-075^T^ | 96.45 |
| RS-N-Gf-1 | *Thalassobius litorarius* MME-075^T^ | 97.62 |
| RS-M-Gl-7 | *Thalassospira profundimaris* WP0211^T^ | 100 |
| RS-M-Gl-3 | *Tritonibacter mobilis* subsp. *pelagius* NBRC 102038^T^ | 99.24 |
| RS-N-Bc-4 | *Tropicimonas isoalkanivorans* DSM 19548^T^ | 98.74 |
| GW-G-Ua-1 | *Vibrio alginolyticus* NBRC 15630^T^ | 99.67 |
| BS-M-Sm-2 | *Vibrio crassostreae* LGP7^T^ | 99.80 |
| RW-H-Ap-7 | *Vibrio tasmaniensis* LMG 21574^T^ | 99.71 |
| RW-H-Av-10 | *Yoonia litorea* DSM 29433^T^ | 98.97 |
| RS-M-Af-20 | *Yoonia litorea* DSM 29433^T^ | 97.44 |
| RW-G-Bc-1 | *Yoonia maricola* DSM 29128^T^ | 98.19 |
| RS-N-Gd-9 | *Yoonia maricola* DSM 29128^T^ | 99.58 |
| RW-H-Cc-7 | *Yoonia rosea* DSM 29591^T^ | 99.57 |
| RW-G-Ge-12 | *Yoonia sediminilitoris* D1-W3^T^ | 99.18 |
| RS-M-Dj-5 | *Yoonia sediminilitoris* D1-W3^T^ | 98.27 |
| GW-G-Ua-6 | *Yoonia sediminilitoris* D1-W3^T^ | 98.11 |
| RS-M-Aa-7 | *Yoonia tamlensis* DSM 26879^T^ | 99.18 |
| **Phylum *Bacteroidota* (57 strains)** | | |
| RS-N-Sl-3 | *Agaribacter marinus* 45877^T^ | 96.12 |
| RW-H-Cc-1 | *Agarivorans albus* MKT 106^T^ | 99.67 |
| RS-M-Ps-2 | *Agarivorans albus* MKT 106^T^ | 99.82 |
| RE-G-Ps-6 | *Agarivorans albus* MKT 106^T^ | 99.37 |
| GE-S-Ul-17 | *Agarivorans albus* MKT 106^T^ | 97.77 |
| RE-S-Sl-7 | *Algibacter aestuarii* KYW371^T^ | 98.08 |
| RW-G-Bc-8 | *Algibacter marinivivus* ZY111^T^ | 97.20 |
| RE-S-Gd-1 | *Algibacter marinivivus* ZY111^T^ | 98.55 |
| RS-N-Gf-3 | *Algibacter mikhailovii* LMG 23988^T^ | 99.91 |
| BS-N-Is-2 | *Aquibacter zeaxanthinifaciens* CC-AMZ-304^T^ | 94.67 |
| RW-H-Gj-23 | *Aquimarina agarivorans* HQM9^T^ | 95.31 |
| GW-H-Cl-5 | *Aquimarina agarivorans* HQM9^T^ | 100 |
| RS-M-Ny-2 | *Aquimarina intermedia* DSM 17527^T^ | 99.70 |
| RS-N-Gd-13 | *Aquimarina latercula* DSM 2041^T^ | 99.31 |
| RE-S-Gd-6 | *Aquimarina latercula* DSM 2041^T^ | 99.49 |
| GW-H-Cl-1 | *Aquimarina latercula* DSM 2041^T^ | 99.52 |
| BE-G-Fc-14 | *Aquimarina latercula* DSM 2041^T^ | 99.47 |
| BS-N-Is-1 | *Aquimarina spongiae* A6^T^ | 100 |
| RW-H-Lh-8 | *Aurantibacter aestuarii* KYW614^T^ | 97.42 |
| RS-N-Sl-10 | *Bizionia echini* DSM 23925^T^ | 97.25 |
| RE-S-Gd-9 | *Cellulophaga pacifica* NBRC 101531^T^ | 94.10 |
| RS-M-Af-2 | *Croceitalea eckloniae* DOKDO 025^T^ | 97.01 |
| RS-N-Gd-16 | *Croceitalea litorea* CBA3205^T^ | 99.90 |
| RW-H-Gj-20 | *Croceitalea marina* H01-35^T^ | 99.60 |
| RW-G-Bg-5 | *Dokdonia donghaensis* DSW-1^T^ | 99.60 |
| RW-G-Af-5 | *Dokdonia donghaensis* DSW-1^T^ | 99.17 |
| BE-G-Fc-4 | *Dokdonia genika* Cos-13^T^ | 97.57 |
| GS-N-Cv-1 | *Flagellimonas algicola* AsT0115^T^ | 96.27 |
| RW-G-Af-17 | *Hyunsoonleella udonensis* JG48^T^ | 98.76 |
| RE-S-Sl-3 | *Lacinutrix mariniflava* AKS432^T^ | 100 |
| RW-G-Bg-3 | *Lacinutrix venerupis* DSM 28755^T^ | 99.40 |
| RW-G-Af-8 | *Maribacter aestuarii* GY20^T^ | 98.74 |
| RS-M-Ny-30 | *Maribacter aestuarii* GY20^T^ | 97.52 |
| RS-M-Ny-7 | *Maribacter arcticus* DSM 23546^T^ | 96.40 |
| RW-H-Cc-9 | *Maribacter chungangensis* CAU 1044^T^ | 98.51 |
| GE-G-Ua-2 | *Maribacter chungangensis* CAU 1044^T^ | 98.45 |
| RW-G-Af-3 | *Maribacter dokdonensis* DSW-8^T^ | 99.90 |
| RS-M-Gl-12 | *Maribacter dokdonensis* DSW-8^T^ | 100 |
| RS-N-Gf-7 | *Maribacter litoralis* SDRB-Phe2^T^ | 94.90 |
| RS-N-Gf-13 | *Maribacter stanieri* DSM 19891^T^ | 97.37 |
| RE-G-Ps-7 | *Maribacter stanieri* DSM 19891^T^ | 98.76 |
| RS-M-Ny-22 | *Muricauda koreensis* ECD12^T^ | 95.98 |
| RS-M-Ps-7 | *Muricauda pacifica* sw027^T^ | 97.19 |
| RW-H-Gj-6 | *Ningiella ruwaisensis* B66^T^ | 94.25 |
| RW-G-Gf-3 | *Nonlabens ulvanivorans* PLR^T^ | 100 |
| RE-S-Sl-4 | *Nonlabens ulvanivorans* PLR^T^ | 99.88 |
| BS-N-Sf-8 | *Olleya algicola* 3Alg 18^T^ | 100 |
| RW-H-Lh-7 | *Polaribacter dokdonensis* DSW-5^T^ | 98.92 |
| RE-S-Sl-10 | *Polaribacter sejongensis* KOPRI 21160^T^ | 98.44 |
| RE-S-Sl-2 | *Wenyingzhuangia aestuarii* MN1-138^T^ | 99.75 |
| RE-G-Bd-9 | *Wenyingzhuangia aestuarii* MN1-138^T^ | 99.76 |
| RS-M-Af-9 | *Winogradskyella echinorum* KMM 6211^T^ | 99.27 |
| RE-S-Gd-15 | *Winogradskyella endarachnes* HL2-2^T^ | 97.41 |
| RE-S-Gd-2 | *Winogradskyella haliclonae* M1A16^T^ | 98.76 |
| RW-H-Lh-1 | *Winogradskyella jejuensis* DSM 25330^T^ | 98.64 |
| RS-M-Gl-5 | *Winogradskyella jejuensis* DSM 25330^T^ | 99.14 |
| RS-M-Aa-2 | *Winogradskyella litoriviva* KMM 6491^T^ | 99.01 |
| **Phylum *Actinomycetota* (3 strains)** | | |
| GS-M-Ul-3 | *Micrococcus flavus* LW4^T^ | 99.63 |
| RW-G-Gf-4 | *Nocardioides cavernae* YIM A1136^T^ | 99.80 |
| RW-G-Bg-2 | *Terracoccus luteus* DSM 44267^T^ | 99.90 |
| **Phylum *Bacillota* (2 strains)** | | |
| GS-M-Ul-7 | *Bacillus infantis* NRRL B-14911^T^ | 99.82 |
| BS-N-Sf-2 | *Ureibacillus chungkukjangi* 2RL3-2^T^ | 100 |

**Table S6** Taxonomic information of representative strains from the genera *Pseudoalteromonas*, *Psychromonas*, *Marinomonas*, and *Litorimonas,* identified as potential keystone taxa of phycospheres based on 16S rRNA gene sequences.

| **Strain** | **Isolation source** | **Closest type strain** | **16S rRNA gene sequence similarity (%)** | **GenBank accession no.** |
| --- | --- | --- | --- | --- |
| RW-H-Ap-1 | *Acrochaetium plumosum* | *Pseudoalteromonas tetraodonis* GFC^T^ | 99.9 | PQ311727 |
| GE-S-Ul-11 | *Ulva linza* | *Psychromonas arctica* DSM 14288^T^ | 99.4 | PQ311728 |
| RS-M-Aa-14 | *Acrochaetium arcuatum* | *Marinomonas algicola* SM1966^T^ | 99.7 | PQ311729 |
| RW-G-Af-16 | *Ahnfeltiopsis flabelliformis* | *Litorimonas taeanensis* DSM 22008^T^ | 96.8 | PQ311730 |

**Table S7** General genomic features^†^ of representative bacterial strains of core taxa in phycospheres.

1, strain RW-H-Ap-1 (CP176466–7); 2, strain GE-S-Ul-11 (CP176468); 3, strain RS-M-Aa-14 (CP176470); 4, strain RW-G-Af-16 (CP176469).

| **Feature**^‡^ | **1** | **2** | **3** | **4** |
| --- | --- | --- | --- | --- |
| No. of chromosome (size, kb) | 2 (3,439; 746) | 1 (3,915) | 1 (4,581) | 1 (2,548) |
| Total genome size (kb) | 4,185 | 3,915 | 4,581 | 2,548 |
| G+C content (%) | 39.9 | 37.9 | 44.1 | 52.6 |
| No. of total genes | 3,906 | 3,451 | 4,262 | 2,396 |
| No. of coding sequences | 3,604 | 3,317 | 4,132 | 2,348 |
| No. of total RNA | 131 | 105 | 111 | 43 |
| No. of tRNA | 101 | 79 | 81 | 37 |
| No. of rRNA (5S, 16S, 23S) | 10, 8, 8 | 8, 7, 7 | 9, 8, 8 | 1, 1, 1 |
| No. of ncRNA | 4 | 4 | 5 | 3 |
| No. of pseudogenes | 171 | 29 | 19 | 5 |
| No. of CAZyme* genes | 60 | 69 | 60 | 39 |
| Glycoside hydrolase | 29 | 18 | 28 | 18 |
| Glycosyltransferase | 17 | 26 | 21 | 10 |
| Polysaccharide lyase | 0 | 14 | 3 | 5 |
| Carbohydrate esterase | 3 | 2 | 2 | 1 |
| Auxiliary activities | 3 | 2 | 2 | 2 |
| Carbohydrate-binding module | 8 | 7 | 4 | 3 |

†The genomic features were analyzed using the NCBI prokaryotic genome annotation pipeline (www.ncbi.nlm.nih.gov/genome/annotation_prok/).

^‡^All genomes were completely sequenced.

*CAZyme, carbohydrate-active enzyme.
